# γ9δ2 T-Cell Expansion and Phenotypic Profile Are Reflected in the CDR3δ Repertoire of Healthy Adults

**DOI:** 10.3389/fimmu.2022.915366

**Published:** 2022-07-07

**Authors:** Anna Vyborova, Anke Janssen, Lucrezia Gatti, Froso Karaiskaki, Austin Yonika, Sanne van Dooremalen, Jasper Sanders, Dennis X. Beringer, Trudy Straetemans, Zsolt Sebestyen, Jürgen Kuball

**Affiliations:** ^1^Center for Translational Immunology, University Medical Center (UMC) Utrecht, Utrecht University, Utrecht, Netherlands; ^2^Department of Hematology, University Medical Center (UMC) Utrecht, Utrecht University, Utrecht, Netherlands

**Keywords:** human γ9δ2 T cells, CDR3δ, adult Vδ2 repertoire, differentiation profile, public clonotypes, NKG2D, CD94/NKG2A(B)

## Abstract

γ9δ2T cells fill a distinct niche in human immunity due to the unique physiology of the phosphoantigen-reactive γ9δ2TCR. Here, we highlight reproducible TCRδ complementarity-determining region 3 (CDR3δ) repertoire patterns associated with γ9δ2T cell proliferation and phenotype, thus providing evidence for the role of the CDR3δ in modulating *in vivo* T-cell responses. Features that determine γ9δ2TCR binding affinity and reactivity to the phosphoantigen-induced ligand *in vitro* appear to similarly underpin *in vivo* clonotypic expansion and differentiation. Likewise, we identify a CDR3δ bias in the γ9δ2T cell natural killer receptor (NKR) landscape. While expression of the inhibitory receptor CD94/NKG2A is skewed toward cells bearing putative high-affinity TCRs, the activating receptor NKG2D is expressed independently of the phosphoantigen-sensing determinants, suggesting a higher net NKR activating signal in T cells with TCRs of low affinity. This study establishes consistent repertoire–phenotype associations and justifies stratification for the T-cell phenotype in future research on γ9δ2TCR repertoire dynamics.

## Introduction

From generation onward, the T-cell receptor repertoire accumulates footprints of selection and clonotypic expansion that reduce its diversity and lead to skewing. As a result, the repertoire of antigen-experienced T cells represents only a modest fraction of the repertoire of their newly generated naïve precursors, limited to the antigenic specificities relevant to the individual’s immunological history [reviewed in (1)]. A γ9δ2TCR is notably different from an αβTCR, as it senses the accumulation of intracellular phosphorylated non-peptide antigens (phosphoantigens or pAgs) through modification of surface butyrophilin family members BTN3A1 and BTN2A1, orchestrated by interaction with the small GTPase RhoB, rather than sensing peptide–MHC complexes (2–6). Nevertheless, despite the unique antigen specificity and recognition mode, the γ9δ2TCR repertoire equally acquires imprints of selection and expansion throughout the human lifetime (7, 8), starting with a wave of polyclonal expansion immediately after birth (9, 10) and ultimately leading to a characteristic “selected,” highly private pAg-reactive γ9δ2TCR repertoire in adults [reviewed in (11)]. While the TCRγ9 chain harbors motifs that are critical for binding the non-polymorphic BTN ligand, the TCRδ2 chain contains the yet unresolved clue to pAg reactivity (4, 12), possibly through the influence of the CDR3δ on the γ9δ2TCR binding affinity in this multiple-ligand system (13, 14). Therefore, the most dramatic repertoire changes are seen in the CDR3δ repertoire from T-cell generation to maturity.

Phenotypic maturation in T cells parallels repertoire maturation and eventually reaches terminal differentiation defined by the dominance of cytotoxic responses over the proliferative, acquisition of natural cytotoxicity markers and reduced TCR signaling (15, 16). γ9δ2T cells mature across a similar continuum of transcriptional, phenotypic, and functional changes as αβT cells do (17, 18). The CD27+CD28+ T cells (“early-stage differentiation profile,” proliferative, characteristic of T cells isolated from cord blood or thymus) or CD27-CD28-CD16+ cells (“late-stage differentiation profile,” readily cytotoxic, seen in selected healthy adults) dominate the two distinct poles of the continuum. Unlike αβT cells, however, γδT cells are characterized already at the fetal stage of T-cell development by a prominent natural killer receptor (NKR) signature, present independently of other differentiation markers (19), and continuing to distinguish γδT cells from their αβ counterparts during adulthood (20, 21).

In early infancy, massive γ9δ2T cell expansion and phenotypic maturation are evidently reflected by enrichment of the γ9δ2TCR repertoire in features that underpin recognition of the pAg-driven changes in BTN2 and BTN3, such as TRGJP rearrangements in the γ chain and a hydrophobic amino acid at position 5 of the CDR3 of the δ chain, suggestive of the key role of these γ9δ2TCR traits in the effector responses of the γ9δ2T cells at that developmental stage (10). As to already highly selected adult γ9δ2TCR repertoires, a clonotypic bias in association with T-cell expansion and differentiation profile has been reported (7); however, it remains unclear whether any distinct TCR features explain this bias. In addition, the expression of certain NKRs has been associated with a CDR3δ bias (22). Considering the growing interest in γδT cells and their TCRs as therapeutics (23), and in reliance on a model in which the γ9δ2TCR diversity is assumed to underpin a range of ligand-binding affinities (13, 14), the question of whether the repertoire changes are related to the phenotypic changes is particularly relevant, since it clears the way for isolating the TCRs with the highest affinity to the antigen and the T-cell populations most potent for therapeutic use (14, 24). In this study, we deconstruct the adult CDR3δ repertoire in relation to the phenotype of the γ9δ2T cells in an attempt to understand the functional role of the γ9δ2TCR diversity in adults.

## Materials and Methods

### Isolation of Peripheral Blood Mononuclear Cells and Cell Phenotyping

Buffy coats were obtained from Sanquin blood bank (Amsterdam, The Netherlands). Peripheral blood mononuclear cells (PBMCs) were isolated from buffy coats using Ficoll-Paque PLUS gradient centrifugation (GE Healthcare, Chicago, IL, USA). The cells at the interface were harvested, washed 4× with phosphate-buffered saline (PBS) (Sigma-Aldrich, St. Louis, MO, USA), and further stained with the following monoclonal antibodies (mAbs): CD3 (CD3-PB Clone UCHT1, BD Biosciences, San Jose, CA, USA; cat. no. 558117), TCR panγδ (TCR panγδ-PE-Cy7 Clone IMMU510, Beckman Coulter, Brea, CA, USA; cat. no. B10247), Vδ2 (TCR Vδ2-FITC Clone B6, BioLegend, San Diego, CA, USA; cat. no. 331406), CD8a (CD8a-PerCP-Cy5.5 Clone RPA-T8, BioLegend; cat. no. 301031), CD4 (CD4-APC Clone RPA-T4, BioLegend; cat. no. 300514), CD27 (CD27-APC-eF780 clone O323, eBioscience, San Diego, CA, USA; cat. no. 47-0279-42) and isotype control (IgG1-APC-eF780 Clone P3.6.2.8.1, eBioscience; cat. no. 47-4714-80), CD45RA (CD45RA-BV650 Clone HI100, BD Horizon, Franklin Lakes, NJ, USA; cat. no. 563963), CD28 (CD28-BV605 Clone CD28.2, BD Horizon; cat. no. 562976), CD16 (CD16-PE Clone eBioCB16, eBioscience; cat. no. 12-0168-41), CD16-BV785 Clone 3G8, BioLegend; cat. no. 302046), CCR7 (CCR7-APC Clone G043H7 Sony Biotechnology, San Jose, CA, USA; cat. no. 2366070), NKG2D (NKG2D-PE clone 1D11, eBioscience; cat. no. 12-5878-42), CD94 (CD94-PE clone HP-3B1, Beckman Coulter; cat. no. IM2276), NKG2A (human CD159a (NKG2A)-APC/Fire™ 750 clone S19004C, BioLegend; cat. no. 375115), and NKG2C (CD159c(NKG2C)-AF700clone 134591, R&D Systems, Minneapolis, MN, USA; cat. no. FAB138N-025).

Live/dead aqua stain (LIVE/DEAD stain kit Aqua fluorescent, Life Technologies, Carlsbad, CA, USA; cat. no. L34957) was used to exclude dead cells.

Cell phenotyping was performed on BD Fortessa and BD Canto, and all fluorescence-activated cell sorting (FACS) processes were performed on BD ARIAII. Sorted T-cell subpopulations were collected in equal numbers in at least duplicates, which were further processed separately as independent biological replicates. We sorted 50,000 cells per population for the late-stage profile donors; for the early-stage donors, there were 12,000–30,000 cells. Cells were collected in ice-cold Roswell Park Memorial Institute (RPMI) medium (Gibco, Grand Island, NY, USA) supplemented with 20% fetal calf serum (FCS) and spun down; cell pellets were resuspended in RLTplus buffer (Qiagen, Valencia, CA, USA; art 1053393) supplemented with fresh β-mercaptoethanol (Life Technologies; art 11528926) (10 µl of βME per 1 ml of RLTplus) for 2 min for lysis at RT and stored at −80°C until RNA isolation.

### TCRδ Chain Sequencing and Data Analysis

RNA isolation, cDNA synthesis, PCR of the TCRδ chain, library preparation, high-throughput sequencing, and analysis of the raw sequencing files were performed at the University Medical Center Utrecht as described earlier (13). In-house R scripts were used for repertoire analysis; data were filtered to include clonotypes with a frequency higher than one read/clonotype. The tcR package (25) was used as the framework of the repertoire analysis pipeline.

All head-to-head comparisons of quantitative repertoire features, such as diversity, were made on samples with equal numbers of sorted cells.

Sequences were annotated as public if they were shared among at least 2 donors in the current study. In order to reduce the uncertainty intrinsic to the detection of low-frequency sequences, which might impair the robust detection of rare public clonotypes, we analyzed only the nucleotypes that had been detected at least in two samples from the same donor. When analyzing public AA clonotypes, multiple (both private and public) nucleotypes encoding for the same AA sequence were collapsed to a single entry with a read count equaling the total of the read counts of all nucleotypes. Several previously published datasets containing high-throughput TCRδ chain sequencing data were used to identify highly common public Vδ2 CDR3 sequences in healthy individuals (9, 10, 26–28).

Analysis of the number of N insertions was performed using the IMGT Junction Analysis tool (29).

### Cells and Cell Lines

Daudi and Phoenix-Ampho cells were obtained from ATCC (Manassas, VA, USA). The TCRβ−/− Jurkat76 cell line was a kind gift from Miriam Heemskerk (LUMC, Leiden, the Netherlands).

### Retroviral Vector Generation

The γ chain of the TCR clone 5 cloned into a pBullet-IRES-neomycin vector *via* 5′ *Nco*I and 3′ *Bam*HI restriction sites was previously available (30). Public and private CDR3δ chain retroviral plasmid vectors were generated based on the previously available retroviral vector encoding the δ chain of the TCR clone 5 (30) by introducing the new CDR3δ sequence using overlap extension PCR as described earlier (13). For the overhang primer pairs encoding the target CDR3δ NT sequences, see **Supplementary Table 3**.

### Retroviral Transduction of T Cells

All public and private TCR δ2 chains were paired with clone 5 TCRγ chain and were transduced into TCR-deficient T-cell line Jurkat76.

For the generation of transduced Jurkat76 cells, Phoenix Ampho cells were transfected with the pBullet retroviral constructs containing TCRγ and TCRδ using FuGENE HD (Promega, Madison, WI, USA). Next, Jurkat76 cells were transduced with the viral supernatants in the presence of polybrene (4 μg/ml; Sigma-Aldrich). The transduced Jurkat76 cells were magnetic-activated cell sorting (MACS)-selected using anti-CD3 microbeads (Miltenyi, Bergisch Gladbach, Germany) and cultured for at least 5 days before functional assay.

### CD69 Upregulation Assay

10^5^ transduced Jurkat76 cells were cocultured overnight in a 1:1 ratio with Daudi cells as a target, with or without 100 µM of pamidronate treatment. The cells were then stained with the following antibody mix for 30 min on ice: Vδ2-FITC clone B6 (BioLegend), CD69-APC clone FN50 (Sony Biotechnology; cat. no. 2154550), and CD20-eFluor450 clone 2H7 (eBioscience; cat. no. 48-0209-42). After being washed, the cells were fixed using 1% paraformaldehyde and analyzed with BD FACS Canto II (BD Biosciences). Readouts were analyzed using FlowJo software.

### Statistical Analysis

Statistical analysis was performed using R Studio. The two-sided Wilcoxon rank-sum test was used to calculate the median difference between two independent groups (31) and its significance.

### Code Availability

Details of the code can be requested from the corresponding author.

## Results

### Clonotypic Expansion Involves Concentration of the CDR3δ Phosphoantigen-Sensing Determinants

As a framework for the study of the repertoire fingerprints of the T-cell expansion, we used the publicly available repertoire data of healthy preterm infants and young children in whom γ9δ2T cells are actively proliferating (10), focusing in this study on the TCRδ chain. Intending to define the CDR3δ features associated with *in vivo* expansion, we classified clonotypes according to their abundance, or clonotype frequency *f* into non-expanded (*f* < 0.1%), expanded (0.1% ≤ *f* < 1%), and hyperexpanded (*f* ≥ 1%) and further analyzed their qualitative traits. Clonotypic expansion associated unambiguously with J1 region usage, and the presence of the “invariant T” nucleotide (invT) (32), or, more generally, a hydrophobic amino acid at position 5 (hAA5) of the CDR3δ (position 109 according to IMGT) (12), features that define the transition from poorly reactive neonatal (8, 19) to adult-type highly pAg-reactive repertoires (7, 9–11, 32) (**Supplementary Figure 1A**). Next to these well-defined pAg-sensing determinants, we noted a marked enrichment in shorter CDR3δ sequences in expanding clones, seen most clearly in the J1-rearranged subrepertoires (**Supplementary Figures 1B, C**). As clonotype frequency might be determined not solely by the expansion of a T-cell clone upon antigen encounter but by generation biases such as short-homology-repeat recombination (33–36) or convergent recombination [reviewed in (11)], we performed a separate analysis of the infant repertoires at the moment of postnatal “γ9δ2T cell burst” (3–5 weeks of age) (10) while splitting the data according to the possible origin of bias. We analyzed independently the germline-encoded sequences (no N insertions) and the sequences with N insertions, thus segregating the effect of the short-homology-repeat recombination. A separate analysis of the sequences shared between the subjects (public sequences) and private sequences allowed to account for the effect of convergent recombination since the generation of most if not all public clonotypes relies on this mechanism [(11); see **Supplementary Table 1**]. Although the highly prevalent “hyperexpanded” clonotypes at this developmental stage are almost exclusively germline-encoded and public, emphasizing the recombination effect on clonotype frequency, enrichment in sequences featuring hAA5 and in shorter sequences with increased clonotype frequency remained equally evident in all independent analyses (**Supplementary Figures 1D, E**), suggesting that both features have a functional implication in T-cell proliferation and do not solely reflect a production bias.

**Figure 1:**
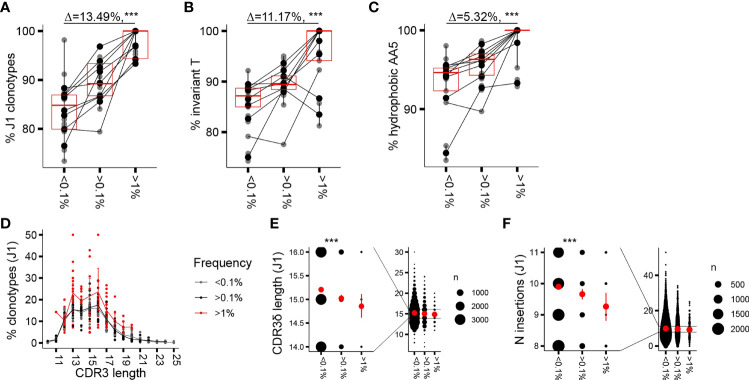
Clonotypic expansion involves concentration of the CDR3δ pAg-sensing determinants. **(A–C)** Proportion of sequences featuring the determinants of pAg reactivity: rearrangement to J1 region **(A)**, the invT **(B)**, and a hAA5 (A/V/L/I/P/W/F/M) **(C)** among clonotypes with increasing *f* in the CD27^pos^ cells. Lines connect the average of the replicates in each donor. Bars represent median values and interquartile range (IQR). The median difference and the p-values were obtained using Wilcoxon rank-sum test. **(D)** CDR3δ length distribution among J1-rearranged clonotypes in the CD27^pos^ cells. Sequences were classified by *f*. Lines connect the mean abundances of clonotypes of each respective length within the frequency category. **(E, F)** CDR3δ length **(E)** and number of N insertions **(F)** in clonotypes rearranged to J1 region. Mean values and error bars representing 95% CI for the mean are shown in red ***P ≤ 0.001 (Wilcoxon rank-sum test).

Similar to the proliferative burst seen in early childhood, a marked γ9δ2T cell expansion associated with loss of CD27 expression has been occasionally noted in adults, resulting in γ9δ2TCR repertoires focused on a few (hyper)expanded clones (7). We wondered whether CDR3δ features that are associated with expansion in infant repertoires similarly underpin clonotypic expansion and T-cell maturation in adults. Therefore, we sorted Vδ2^pos^CD27^pos^ and Vδ2^pos^CD27^neg^ populations in a cohort of 9 adult donors (**Supplementary Figure 2A**) and performed high-throughput sequencing of the TCRδ chain of the sorted subsets, first focusing the analysis on the phenotypically younger CD27^pos^ cells. The gating strategy at sorting did not include an antibody directed against the Vγ9 chain (**Supplementary Figure 2A**); however, we confirmed that Vγ9^neg^Vδ2^pos^ cells represented less than 3.5% (median 1.75%) of the total Vδ2^pos^ sorted cells and less than 10% (median 6.8%) of the Vδ2^pos^CD27^pos^ subset. Vγ9^neg^Vδ2^pos^ cells were mainly Vδ2^dim^ cells, a population gated out during sorting (**Supplementary Figure 2B**). As seen with the infant and the child repertoires, in the CD27^pos^ cells of the healthy adults, clonotype frequency was positively associated with rearrangement to the J1 region, the invT nucleotide, and the hAA5 (**Figures 1A–C**); however, the effect sizes observed were smaller in comparison to changes seen in early ontogeny. Within the dominant subset of clonotypes rearranged to the shorter J1 region, a far less notable but still discernible enrichment in shorter sequences was seen with increased clonotype frequency, largely due to the fact that none of the long (>20 AA) sequences reached *f* of 1% (**Figures 1D, E**). Not surprisingly, as parts of the variable stretch between the conserved motifs of the CDR3δ are encoded by N nucleotides, we saw a similar decrease in the average number of the N insertions (**Figure 1F**). Germline-encoded sequences and public sequences are fewer in number and occupied space in adult repertoires compared to the infant ones (**Supplementary Figures 2C, D**); nevertheless, we repeated the analysis of private subrepertoires and subrepertoires generated with N insertions to account for the possible generation bias in these phenotypically “young” cells. We obtained overall similar trends for enrichment in J1 rearrangements, invT, and hAA5 (**Supplementary Figure 2E**) and equally in the shorter sequences, despite an overall shift toward slightly greater mean CDR3 lengths after sorting out the public sequences (**Supplementary Figures 2F, G**; see **Figure 3** and the corresponding text for the characteristics of the public sequences).

Thus, CDR3δ features postulated as determinants of sensing pAg-driven changes in BTN2 and BTN3 likely underpin the *in vivo* clonotypic expansions seen in infancy and adulthood, although the enrichment patterns are less pronounced in adult repertoires that are nearly saturated with pAg-reactive clones. Clonotypic expansion is further associated with a narrowing range of CDR3δ lengths. Enrichment in shorter sequences in expanded clones, translating, in part, from fewer N insertions, is readily apparent at the time of active T-cell expansion in infants and, as a reminiscence of the patterns seen in infancy, is still discernible in the repertoires of CD27^pos^ cells in adults.

### Phenotypic Maturation Reflects Repertoire Focusing, Which Is Independent of the CDR3δ Traits

**Figure 2:**
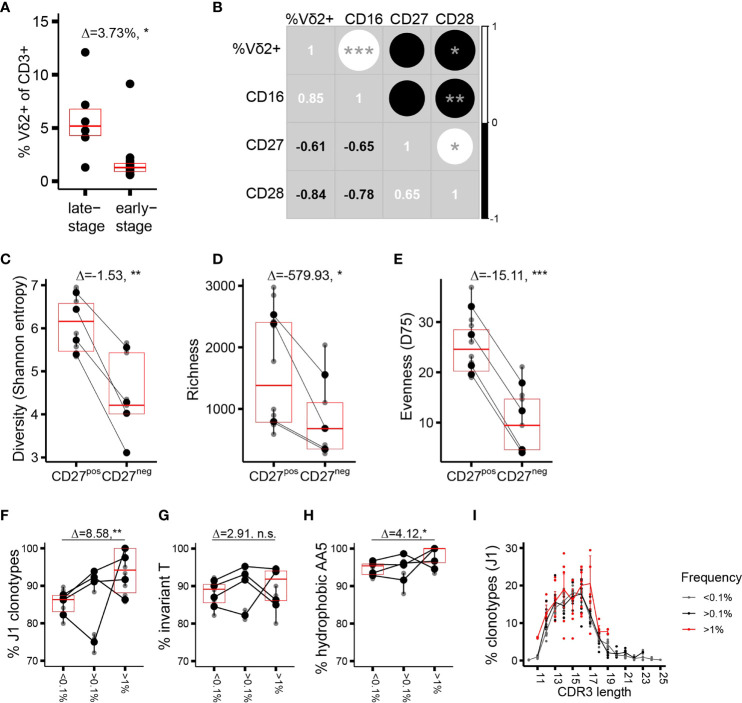
Phenotypic maturation reflects repertoire focusing which is independent of the CDR3δ traits. **(A)** Proportion of Vδ2TCR+ T cells among CD3+ cells in the donors of different phenotypic profiles. **(B)** Correlation matrix showing Spearman’s correlation coefficients for the proportion of Vδ2+ T cells among CD3+ T cells, and the proportion of CD27+, CD28+, or CD16+ T cells among Vδ2+ T cells, measured in an independent set of 10 healthy donors. **(C–E)** Measures of diversity: Shannon entropy **(C)**, repertoire richness (number of individual clonotypes, **(D)**, and repertoire evenness, defined as D75 **(E)** in the late-stage profile donors (n = 4). **(F–H)** Proportion of sequences featuring the determinants of pAg reactivity: rearrangement to J1 region **(F)**, the invT **(G)**, and a hydrophobic AA5 **(H)** among the clonotypes with increasing frequency *f* in the CD27^neg^ cell population of the late-stage donors. Bars represent median values and IQRs. The median difference and the p-values were determined using Wilcoxon rank-sum test. *p < 0.05; **p ≤ 0.01; ***p ≤ 0.001. **(I)** CDR3δ length distribution among J1-rearranged clonotypes in the CD27^neg^ cells. Sequences were classified by *f*. Lines connect the mean abundances of clonotypes of each respective length within the frequency category.

We next focused on the repertoire traits associated with phenotypic maturation. Assuming that the phenotype shifts result from an antigenic trigger in the periphery (37), we thus aimed to identify CDR3δ traits underlying peripheral repertoire selection. As adult donors are extremely heterogeneous with respect to the proportions of cells at distinct stages of differentiation (17), we concentrated on the part of the cohort in which a sizable fraction of the Vδ2+ compartment has completed differentiation to CD27−CD28−CD45RA++CD16+ phenotype, designated here as late-stage differentiation profile donors (n = 6 of the 20 donors phenotyped, cells from 4 out of 6 donors sorted, and repertoires sequenced; see **Supplementary Figure 2A**, an example of a healthy donor (HD) 94, and **Supplementary Figure 3A**). The rest of the cohort (n = 14, cells from 5 donors sorted and repertoires sequenced) was collectively characterized by the dominance of CD27^pos^ cells (**Supplementary Figure 2A**, example of HD101), as well as by a less-differentiated state of the sorted CD27^neg^ population, and was categorized here as belonging to the early-stage differentiation profile (**Supplementary Figure 3A**). Late-stage profile donors had a higher proportion of Vδ2TCR+ T cells among CD3+ cells (**Figure 2A**), and the expression of each differentiation marker in the cohort correlated positively (CD16) or negatively (CD27, CD28) with the percentage of Vδ2TCR+ T cells among total T cells (**Figure 2B**). The CD27^neg^ population showed a markedly lower CDR3δ repertoire diversity (estimated here using Shannon entropy) compared to the CD27^pos^ cells (**Figure 2C**). This resulted from a lower number of clonotypes in the CD27^neg^ subset, suggesting a selection process as cells mature from “young” CD27^pos^ to terminally differentiated CD27^neg^ phenotype (**Figure 2D**), as well as from lower evenness, indicating a greater degree of clonotypic expansion in the terminally differentiated cells (evenness was estimated using D75, the percentage of unique clonotypes that occupy 75% of the sample library, **Figure 2E**). The same metrics in early-stage profile individuals showed less profound and less consistent demarcation between the populations sorted according to CD27 expression (**Supplementary Figures 3B–D**). In general, cells with larger distances in phenotype showed more CDR3δ repertoire dissimilarity. Thus, the TCRδ repertoire in phenotypically differentiated cells is shaped by both selection of clonotypes and clonotypic expansion, the latter arguably effectuating the observed inflation of the Vδ2+ compartment as a whole in the late-stage profile donors.

We then analyzed the qualitative repertoire traits that could denote repertoire selection, performing first a head-to-head comparison of the entire repertoires of the sorted subsets. Only a marginal enrichment in the pAg reactivity determinants (**Supplementary Figures 4A–C**) a slight shift towards longer CDR3 length (**Supplementary Figure 4D**) and no change in the number of N insertions (**Supplementary Figure 4E**) characterized the transition from CD27^pos^ to CD27^neg^ phenotype at the level of the complete repertoires. Considering the extreme focusing in the CD27^neg^ compartment (median D75 of 9.43%), we were particularly interested in the characteristics of the high-frequency clonotypes. The “hyperexpanded” repertoires of the CD27^neg^ cells appeared to be permissive to occasional J3 rearrangements, and sequences featuring “non-invT encoded” amino acids, mostly alanine and glycine (**Figures 2F–H**). Further, the CDR3δ length distribution that was centered around the 13 and 16 AA in the CD27^pos^ subset here peaked at 14 and 17 AA (**Figure 2I**). Thus, although terminally differentiated cells harbor a TCR repertoire slightly more “saturated” in sequences sensing the pAg-driven ligand than cells of CD27^pos^ phenotype, the extreme clonotypic expansions that skew the CDR3δ repertoires in the late-stage donors to near oligoclonality do not strictly rely on the pAg-sensing determinants, and therefore non- or poorly pAg-reactive T-cell clones may take prominent positions in the repertoire.

### Public Delta Clonotypes Expand *In Vivo*, But Their Number Diminishes Upon Maturation

**Figure 3:**
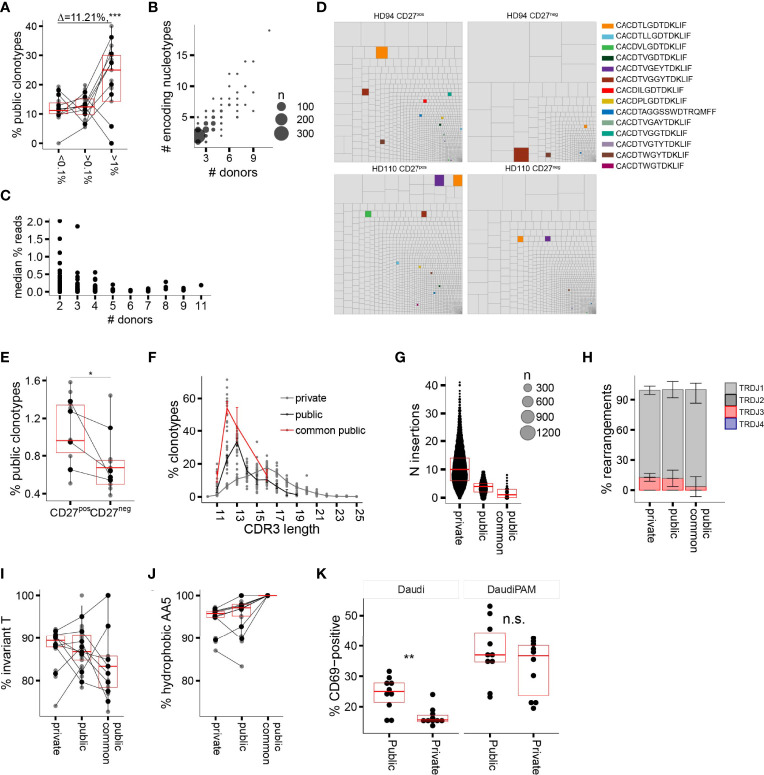
Public delta sequences: persistence in the adult repertoire, qualitative traits, and functionality. **(A)** Percentage of public AA CDR3δ sequences among clonotypes with increasing *f*. **(B)** Number of individual nucleotypes encoding for individual public AA sequence in relation to the sequence “publicity” (number of individual donors sharing a given sequence). **(C)** The relationship between the number of donors sharing a public sequence and the median *f* (all donors pooled together; each dot represents an individual sequence). **(D)** Representative treemaps of the repertoires of CD27^pos^ and CD27^neg^ populations of two late-stage profile donors. Public AA clonotypes are highlighted in color; private sequences are shown in gray. **(E)** Percentage of highly common public AA CDR3δ sequences in the repertoires of CD27^pos^ and CD27^neg^ populations in the late-stage profile donors (n = 4). **(F)** CDR3δ length distribution of private vs. public AA sequences; the highly common public clonotypes are shown separately in red. **(G)** Number of total N insertions in private vs. public AA sequences. **(H–J)** J region usage **(H)**, invT **(I)**, and hAA5 **(J)** in private versus public AA sequences. **(K)** Percentage CD69^pos^ TCR-transduced Jurkat76 cells upon overnight incubation with target cell line Daudi without or in the presence of 100 μM of PAM. In all panels horizontal bars represent median values and IQRs. The median differences and the p-values were determined using Wilcoxon rank-sum test. *p < 0.05; **p ≤ 0.01; ***p ≤ 0.001. Panels **(A, C, F–J)** refer to the CD27^pos^ cell populations.

CDR3δ clonotypes shared between unrelated subjects (public δ clonotypes) have been shown to play a prominent role in the first wave of postnatal expansion of the γ9δ2T cells in newborns (9, 10). However, their number decreases with advancing age in children (10) and decreases even further in adults, possibly as a result of peripheral repertoire selection (7, 27), changes in thymic output after birth (36), or both. Here we aimed to explore the expansion potential and the evidence for peripheral selection of the public sequences in adults, by tracing the sequences shared between at least two individual donors in the current study. The proportion of public clonotypes increased with increasing *f* in the CD27^pos^ population in the majority of the donors (**Figure 3A**). More widely shared clonotypes were encoded by larger numbers of nucleotypes, emphasizing that convergent recombination is the foundation of publicity (**Figure 3B**); however, we detected no tendency toward more common clonotypes having higher frequencies (**Figure 3C**), unlike the observations made for the public sequences of fetal origin in infants (10). We then narrowed the analysis down to public sequences with a high degree of sharing, i.e., sequences detected in >50% of the donors included in this study and present in multiple published studies (**Table 1**; see **Supplementary Table 1** for the encoding nucleotypes). We looked at the impact of peripheral repertoire selection on the maintenance of the public sequences, again using the model of transition from the CD27^pos^ phenotype to terminal differentiation in the late-stage profile donors. We observed a loss of the highly common public sequences as the cells evolve from CD27^pos^ to the CD27^neg^ phenotype (**Figures 3D, E**), suggesting their deletion in the periphery as cells mature. Thus, public TCRδ clonotypes persist in the repertoires of healthy adults and expand *in vivo* but are only partly maintained when cells undergo terminal differentiation.

**Table 1:** Public clonotypes shared among >50% of donors in this study and their occurrence in other published repertoire studies.

Public CDR3δ clonotypes	CDR3δ length	Nucleotypes		# donors in this study	Adult repertoires			Infant and child repertoires	
		Public	Private		(27)	(26)	(28)	(10)	(9)
CACDTLGDTDKLIF*	12	6	13	11	+	+	+	+	+
CACDTLLGDTDKLIF	13	5	9	9	+	+	−	+	+
CACDVLGDTDKLIF*	12	2	4	9	+	+	+	+	+
CACDTLGVYTDKLIF	13	1	8	9	−	−	+	+	+
CACDTVGDTDKLIF*	12	2	8	8	+	+	+	+	+
CACDTVGEYTDKLIF	13	2	5	8	+	+	+	+	+
CACDTVGGYTDKLIF	13	3	11	8	+	+	−	+	+
CACDILGDTDKLIF	12	2	3	7	+	+	+	+	+
CACDPLGDTDKLIF	12	2	10	7	−	+	+	+	+
CACDTAGGSSWDTRQMFF	16	2	4	7	−	+	+	+	+
CACDTVGGTDKLIF	12	3	6	7	−	+	+	+	−
CACDTVGTYTDKLIF	13	0	8	7	+	+	+	+	−
CACDTWGTDKLIF	11	1	6	7	+	+	+	+	+
CACDTWGYTDKLIF*	12	2	3	7	+	+	+	+	+

Sequences used for the TCR transfer experiment (see below) are marked with an asterisk.

### Public Delta Clonotype Function: The Beauty of Brevity

The published evidence for the expansion of public Vδ2 clonotypes in infants (10), our current data on their persistence into adulthood, the high degree of sharing and *in vivo* proliferation in adults made us wonder whether these sequences may share properties that would confer high TCR affinity to the pAg-driven ligand. We first characterized the qualitative features of adult public CDR3δ sequences, looking for distinct traits that could potentially underpin their functionality. Public sequences were most notable for their short average length, which peaked at 12AA for the most common public clonotypes versus 16AA for the private sequences (**Figure 3F**), and the underlying low number of N insertions (**Figure 3G**). Public and private sequences did not differ in J region usage (**Figure 3H**), while the CDR3δ AA5 was exclusively hydrophobic in common public sequences, with relative enrichment in non-invT-encoded amino acids A and W (**Figures 3I, J**).

In order to assess the impact of the public and private TCRδ chain properties on TCR functionality, we selected four common public CDR3δ sequences present in this study, as well as in previously published repertoire studies (9, 10, 26–28) (**Table 1**), which all have 12AA length, and expressed them alongside a fixed γ chain of the TCR clone 5 (30). A set of private sequences of various lengths (median length 16AA), all rearranged to the J1 region and all harboring a hAA5, was taken along as a control (**Supplementary Table 2**). All constructs were transduced into TCR-deficient Jurkat76 (J76) cells, yielding similar γδTCR surface expression levels (**Supplementary Figure 5A**), and CD69 upregulation by J76 cells upon co-culture with the tumor cell line Daudi, was used as a marker for TCR-mediated T-cell activation. We saw higher T-cell activation with the short public δ sequences in the condition without N-amino bisphosphonate (NBP), while NBP treatment eliminated the difference (**Figure 3K**). There was no confounding effect of the TCR surface expression level on the observed differences (**Supplementary Figure 5B**). In summary, a shorter length of public CDR3δ sequences, in addition to the consistent positivity for hAA5, is associated with a higher functional avidity, as demonstrated in the TCR gene transfer experiment.

### Surface NKG2D Expression Declines With T-Cell Maturation and Is Independent of TCR Clonality

**Figure 4:**
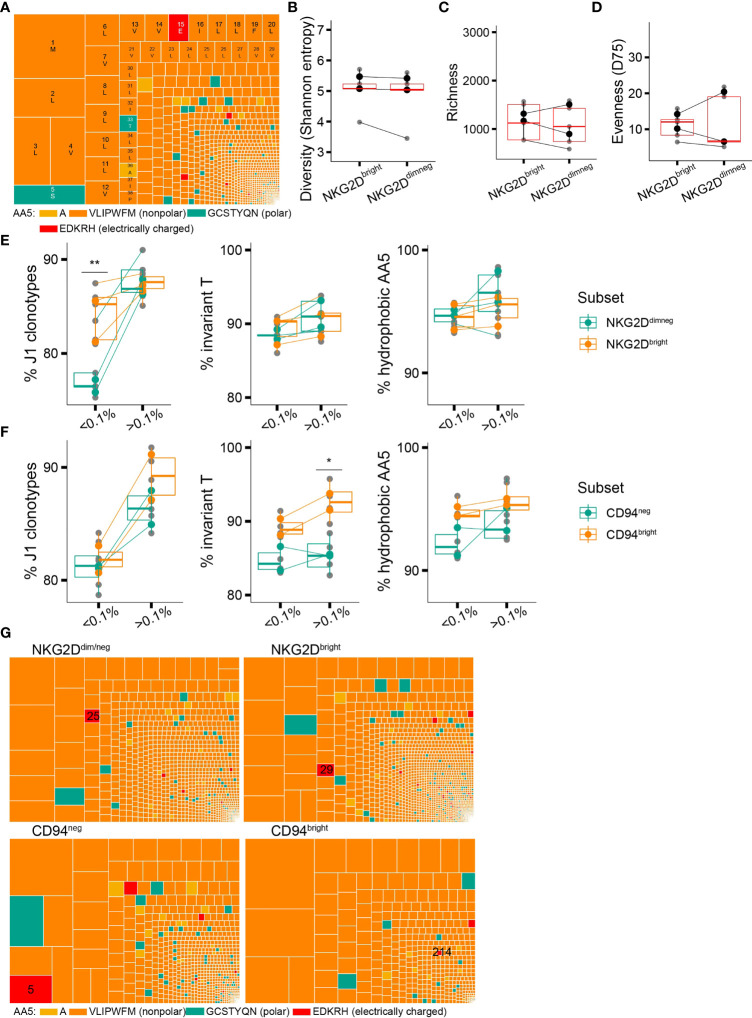
Non-clonally restricted NKG2D expression versus a CDR3δ bias in surface expression of CD94/NKG2A(B). **(A)** An example of a repertoire of a healthy donor where a putative non-pAg-reactive clonotype occupies a prominent position (position 16, CACDT**E**GTPTLLIF). **(B–D)** TCR repertoire diversity of the NKG2D^dim/neg^ and NKG2D^bright^ cell populations estimated with Shannon entropy **(B)**, species richness **(C)**, and evenness(D75) **(D)** (n = 3 donors). y-Axis limits are set equal to those in **Figures 2C–E**. **(E)** Qualitative features of the repertories of the NKG2D^dim/neg^ and NKG2D^bright^ cells in relation to clonotype frequency: proportion of sequences rearranged to J1 region and proportion of sequences featuring the invT or a hAA5. **(F)** Qualitative features of the repertories of the CD94^neg^ and CD94^bright^ cells in relation to clonotype frequency: proportion of sequences rearranged to J1 region and proportion of sequences featuring the invT or a hAA5 (n = 2 donors). **(G)** Treemaps showing the CDR3δ repertoires of the NKG2D^dim/neg^, NKG2D^bright^, CD94^bright^, and CD94^neg^-sorted populations in the same healthy donor as in **(A)** Position of the putative non-pAg reactive clonotype CACDT**E**GTPTLLIF is indicated. **(B–F)** Bars represent median values and interquartile range (IQR) (Wilcoxon rank-sum test, *p < 0.05; **p ≤ 0.01; ***p ≤ 0.001).

Despite the general trend for preferential *in vivo* expansion of putative high-affinity CDR3δ clones containing the traits associated with sensing pAg-driven changes in BTN2 and BTN3, we occasionally witnessed (hyper)expanded clonotypes harboring polar, or even charged amino acids at position 5 of the CDR3δ (**Figure 4A**), which according to the existing experimental evidence are non- or poorly pAg-reactive (13, 14). We hypothesized that pAg-unrelated stimuli could trigger *in vivo* proliferation of such T-cell clones and that poorly pAg-reactive TCRs could possibly benefit from the expression of non-clonally restricted activating receptors. In exploring this hypothesis, we focused on NKG2D, the best-studied activating NKR on γδT cells, previously reported to play both an autonomous stimulatory role (38–40) and a co-stimulatory role next to a γδTCR (41, 42).

We found the absolute majority of the Vδ2+ T cells in adult blood to express NKG2D (**Supplementary Figure 6A**). NKG2D expression correlated with T-cell differentiation phenotype within a donor, as, in general, more differentiated CD27^neg^CD28^neg^ T cells expressed less surface NKG2D (**Supplementary Figure 6B**). The trend for declining surface expression with T-cell maturation was in line with lower NKG2D mRNA expression levels in more differentiated populations, detected in an independent dataset that we extracted from the study by Ryan et al. (17) (**Supplementary Figure 6C**). Conversely, gating on NKG2D^bright^ vs. NKG2D^dim/neg^ cells (**Supplementary Figure 6D**) revealed an overall “younger” phenotype of the NKG2D^bright^ cells in the majority of donors, with notably higher expression of CCR7, CD27, and TCRγδ (**Supplementary Figures 6E, F**), with the NKG2D^intermediate^ population lying between two extremes (**Supplementary Figures 6G, H**).

To examine the repertoire characteristics of Vδ2+ T cells in the context of NKG2D expression, we sorted NKG2D^dim/neg^ and NKG2D^bright^ Vδ2+ T cells as shown in **Supplementary Figure 6D** and again performed sequencing of the TRDV2 repertoires as described above. Unlike the T-cell differentiation profile, the NKG2D expression level did not denote any change in the TCR diversity metrics (**Figures 4B–D**). When we looked into the set of qualitative CDR3δ features that could define the unique clonotypes, we detected no difference between the NKG2D^neg/dim^ and NKG2D^bright^ cells, except for a bias in J1 region usage in the low-frequency clones (**Figure 4E**), which, however, did not translate into a shorter average CDR3δ length (**Supplementary Figure 6I**).

Thus, although the expression level of the activating receptor NKG2D in adult γ9δ2T cells shows a discernible relation to the T-cell differentiation status, it is not associated with a bias toward putative low-affinity or pAg-unreactive TCRs.

### Expression of Inhibitory NKR CD94/NKG2A(B) Associates With a Bias Toward Putative High-Affinity γ9δ2TCRs

The net total of the inhibitory and activating signals from the environment will direct the γ9δ2T cell effector functions (43). Although we did not find compelling evidence for skewing in NKG2D expression toward putative pAg-unreactive clones, there could be still an effect of the signal strength through the γδTCR, generally considered “signal one” (44), on the landscape of the remaining NKRs and thus on the net NKR signal. We therefore aimed to investigate the TCR repertoire features in the context of surface expression of CD94/NKG2A(B), an inhibitory C-type lectin receptor broadly expressed on the γ9δ2T cells and recently reported to identify a highly pAg- and tumor-reactive γ9δ2T cell subset (45).

We sorted Vδ2+ T cells from peripheral blood based on CD94 expression into a CD94^bright^ subpopulation, in which CD94 is known to heterodimerize with NKG2A(B) (22, 46), and a CD94^neg^ subpopulation (**Supplementary Figures 6J, K**), and performed a similar comparative CDR3δ repertoire analysis. CD94^bright^ cells were collectively characterized by a higher percentage of sequences featuring an invT-encoded or generally hydrophobic AA5 in the repertoire (**Figure 4F**), in line with an earlier low-throughput sequencing study (22). CDR3δ bias according to the invT/hAA5 as one of the strong predictors of the TCR reactivity is further exemplified here by the differential clonotypic expansion of a single pAg-unreactive clonotype (**Figure 4G**). Moreover, we observed a trend toward enrichment in shorter CDR3δ sequences in CD94^bright^ cells (**Supplementary Figure 6L**).

In summary, the CDR3δ features known to underpin pAg-sensing and γ9δ2TCR signal strength appear to impact the surface expression of an inhibitory NKR CD94/NKG2A(B), with the putative high-affinity CDR3δ sequences being accompanied by high CD94/NKG2A(B) surface expression, while low-affinity TCR-bearing clones remained predominantly CD94-negative.

## Discussion

The discovery of the germline region-mediated interactions at the basis of the mechanism of antigen recognition by a γ9δ2TCR (2, 4) provides a rationale for the observed *in vitro* (47) and *in vivo* (9, 10) polyclonal γ9δ2T cell responses to antigenic stimuli, fostering the paradigm of the γ9δ2TCR as a pattern recognition receptor. The absence of a known polymorphic ligand leaves the role of the highly diverse CDR3 regions currently undefined. Nevertheless, particularly in the case of the CDR3δ repertoire, a distinct evolution pattern seen during ontogeny (11) hints at the still significant role of the CDR3δ in antigen recognition. Here we expand the evidence on the role of CDR3δ in γ9δ2TCR-mediated responses. We show that *in vivo* γ9δ2T cell expansion and phenotypic maturation are associated with repertoire enrichment in the CDR3δ determinants of sensing pAg-induced changes (12, 48, 49), presumably equating to the concentration of the γ9δ2TCRs with higher binding affinity and signal strength (13, 14). We find that the number of the highly common public TCRδ sequences that combine all known pAg reactivity features, known to diminish with the transition from infant to adult repertoires, diminishes even further within an individual upon T-cell maturation, despite optimal signaling. Lastly, we detect skewing in the inhibitory NKR CD94/NKG2A(B) surface expression toward cells expressing putative high-affinity TCRs. In contrast, expression of the activating NKR NKG2D emerges as independent of CDR3δ traits, suggesting possibly higher net NKR activating signal in T cells with low γ9δ2TCR affinity.

Prevalence of a clonotype in the repertoire, especially in the repertoires of infants and of the “younger” CD27^pos^ cells in adults, had the most apparent association with the features of the Vδ2 chain known as “sensors” of the pAg-induced changes in the target. Such features include rearrangement to the J1 region, a hydrophobic AA5, and, notably, an optimal (shorter) CDR3δ length. Although generation biases such as short-homology-repeat recombination (33–36) and convergent recombination (11) play a role in shaping the repertoire, especially that of the immature cells, the above-described enrichment patterns remained evident in the analysis of subrepertoires generated by either mechanism, suggesting the true implication of the pAg-sensing features in T-cell proliferation, and not solely a reflection of a generation bias. The enrichment was less pronounced in adult repertoires, compared to the repertoires of infants, presumably as a result of the switch from the fetal-type to adult-type thymic output (36), which is nearly saturated with pAg-reactive clones.

Analysis of the CD27^pos^ and CD27^neg^ populations in the late-stage profile donors allowed us to correlate distances in phenotype to CDR3δ repertoire dissimilarity. A change in phenotype from young CD27^pos^CD28^pos^ cells to a mature CD27^neg^CD28^neg^CD16^pos^ profile was associated with extreme repertoire focusing, suggesting a major proliferation event preceding maturation, or, alternatively, expansion of the already mature cells (20). Such inflation remained visible at the level of the entire Vδ2 compartment in these donors, and both the visible inflation and mature phenotypes of these cells suggested preceding antigenic triggers rather than possible generation bias. Although the enrichment in putative high-affinity clonotypes was equally evident with increasing clonotype frequency in the mature CD27^neg^CD28^neg^CD16^pos^ cells, the hyperexpanded clonotypes in the terminally differentiated subpopulation occasionally encompassed sequences that in theory mediate submaximal pAg reactivity.

Earlier studies of the αβT cells highlighted the mechanistic differences between homeostatic proliferation with the maintenance of the T-cell memory phenotype that depends on the strength of tonic TCR signaling, in comparison to massive expansion with differentiation to the effector phenotype as a result of antigenic stimulation [reviewed in (37)]. We suggest the possibility of a similar disparity here: while expansion in the “young” (naïve and central memory) cells, defined here as CD27^pos^, clearly showcases the enrichment in the CDR3δ traits associated with TCR binding affinity, the repertoire of the terminally differentiated CD27^neg^ population might reflect additional imprints of the stimuli other than BTNs/pAgs, which bypass signaling through the TCR. Another argument supporting the hypothesis of critical (co-)stimulants involved in massive oligoclonal proliferation seen in late-stage profile donors is the maintenance of the once-established profile over long time periods, even under repetitive NBP stimulation (17). Likewise, *in vitro* stimulation with pAg or NBPs could not induce such massive oligoclonal outgrowth from PBMCs (47); however, the latter study did not specify the phenotype of the cells used for stimulation experiments.

Sequences that universally possess the key pAg reactivity features are the (near-)germline public TCRδ sequences of high generation probability (50) shared between the repertoires of multiple unrelated individuals. The repertoire studies published to date (7–10, 27, 28, 36, 47) define public sequences at the AA rather than at the NT level, to highlight the functionality of a sequence rather than its generation probability, and to account for convergent recombination (11). Thus, public Vδ2 clonotypes have recently been shown to expand *in vivo* shortly after birth (9, 10) and *in vitro* after stimulation with NBP of cord blood-derived γ9δ2 T cells (47). The more common sequences appear to take more prominent positions in the infant repertoires, pointing to the functional relevance of the public sequences at this stage of development (10). Here we found highly common sequences seen in children across continents, back in the adult repertoires, whether due to persistence from early childhood or *de novo* thymic synthesis at a later stage. The more common sequences had a higher number of encoding individual nucleotypes, emphasizing the role of convergent recombination in sequence sharing. However, we did not find the most common public sequences to occupy more repertoire space in adults, suggesting that recombination bias, while explaining occurrence in multiple unrelated donors, does not necessarily govern repertoire focusing in an individual donor. Being uniformly positive for a hAA5, and except for an occasional J3-rearranged sequence distinguished by a short length of 11-13 AA, the highly common public δ sequences appeared to confer higher functional avidity when expressed alongside a fixed γ chain, as demonstrated by the TCR transfer experiment, suggesting their functional advantage. However, high-dose pamidronate treatment minimized the difference between the shorter public δ chains and the longer private ones, supposedly through conjugation enhancement and stabilization of the immunological synapse in case of putative low-affinity TCRs (13). Remarkably, as the cells differentiate to effector phenotype in the periphery, public sequences are partly disappearing. Thus, a decline in the number of public clonotypes set by the switch in thymic production from the neonatal to adult-type, more private output (36), is extended further, due to negative selection in the periphery.

Collectively, our results support the role of the specific features of the CDR3δ as a γ9δ2TCR signaling modulator by demonstrating their steady enrichment associated with clonotypic expansion, and to a lesser extent with the peripheral repertoire selection *in vivo*. In addition, our results corroborate the paradigm of the orderly evolution of the CDR3δ repertoire, rather than its use for random barcoding. *In vivo* T-cell homeostatic proliferation is related to J region usage, hAA5, and, no less critically, CDR3δ length. At the same time, antigen-driven outgrowth might result in the endorsement of putative low-affinity sequences at the prominent positions in the repertoire and the loss of the high-affinity public sequences.

The expression of NK receptors on terminally differentiated CD8+ αβT cells has been associated with defective TCR signaling and a shift from TCR-mediated to NKR-mediated effector functions, including NKG2D-mediated responses (15, 16, 51). Surprisingly, in the majority of the donors, we found NKG2D expression to decline as γ9δ2T cells mature. NKG2D surface expression could not point to clonotypes with supposed weak γ9δ2TCR signaling, substantiating the notion of its constitutive expression that is uncoupled from the γ9δ2TCR signal strength. In contrast, we observed a bias toward high-affinity CDR3δ features in the surface expression of CD94/NKG2A(B), corroborating the results of the earlier low-throughput study (22). The skewed expression of CD94/NKG2A(B) found here is in line with the activation-inducible expression of this inhibitory NKR in CD8+ αβT cells (52) and its low expression on γ9δ2T cells at the early stages of development, compared to relatively high NKG2D expression already *in utero* (19). We consider the observed CDR3δ bias a likely explanation for the recently reported high antitumor responses of the NKG2A^pos^ γ9δ2T cells (45).

From the translational standpoint, with the advancing field of γδT cell- and γδTCR-based therapies in mind, the repertoire–phenotype associations explored in this study are crucial. Researchers exploring these areas should consider the fact that donors with the highest numbers of γ9δ2T cells in the periphery, which are likely to be selected for *in vitro* experiments, as it is easy to get sufficient cell numbers, will frequently harbor cells whose phenotype is largely skewed toward terminal differentiation and whose repertoire, in turn, will be skewed to near-oligoclonality, where potentially poorly pAg-reactive clonotypes may take prominent positions. Concerning the design of the high-affinity γ9δ2TCR-based therapies (14, 24), CDR3δ features such as the AA5 and CDR3 length are essential, while J1 region usage might reflect an advantage of the shorter length. Lastly, as a high-affinity γ9δ2TCR will influence the net NKR signaling through upregulation of inhibitory receptors, it is worth modifying this second signal in favor of the activating arm.

## Data Availability Statement

FASTQ files of the TRD sequences are deposited and available under the Sequence Read Archive (SRA) accession code PRJNA851385 (http://www.ncbi.nlm.nih.gov/bioproject/851385).

## Ethics Statement

Ethical review and approval was not required for the study on human participants in accordance with the local legislation and institutional requirements. Written informed consent for participation was not required for this study in accordance with the national legislation and the institutional requirements.

## Author Contributions

AV, AJ, DB, and JK designed the experiments. AV, AJ, LG, AY, SD, and FK performed the experiments and analyzed the data. AV, AJ, and JS generated and analyzed the high-throughput sequencing data. AV and JK wrote the manuscript. DB, TS, ZS, and JK supervised the work. All authors provided critical reviews.

## Funding

Funding for this study was provided by ZonMW 43400003 and VIDI-ZonMW 917.11.337, KWF UU 2010-4669, UU 2013-6426, UU 2014-6790, and UU 2015-7601, UU 2018-11393, UU 2018-11979, UU 2020-12586, and UU 2021-13043 to JK.

## Conflict of Interest

JK reports grants from Gadeta, Novartis, and Miltenyi Biotech and is the inventor of patents dealing with γδT cell-related aspects, as well as the co-founder and shareholder of Gadeta. AV, AJ, DB, and ZS are inventors of patents dealing with γδT cell-related aspects.

The remaining authors declare that the research was conducted in the absence of any commercial or financial relationships that could be construed as a potential conflict of interest.

## Publisher’s Note

All claims expressed in this article are solely those of the authors and do not necessarily represent those of their affiliated organizations, or those of the publisher, the editors and the reviewers. Any product that may be evaluated in this article, or claim that may be made by its manufacturer, is not guaranteed or endorsed by the publisher.
