# Supplementary material for: γ9δ2 T-Cell Expansion and Phenotypic Profile Are Reflected in the CDR3δ Repertoire of Healthy Adults
Source: Front Immunol. 2022 Jul 7;13:915366. doi: 10.3389/fimmu.2022.915366 (PMC9301380; doi:10.3389/fimmu.2022.915366)
Supplement: Supplementary file 1 [file DataSheet_1.docx]

Supplementary Information for

In vivo expansion and phenotypic profile of the γ9δ2 T cells are reflected in the Vδ2 TCR repertoire of healthy adults.

Anna Vyborova, Anke Janssen, Lucrezia Gatti, Froso Karaiskaki, Austin Yonika, Sanne van Dooremalen, Jasper Sanders, Dennis X. Beringer, Trudy Straetemans, Zsolt Sebestyen, Jürgen Kuball

Corresponding author: Jürgen Kuball

Email: J.H.E.Kuball@umcutrecht.nl<mailto:xxxxx@xxxx.xxx>

**This file includes:**

Figs. S1 to S6

Tables S1 to S3

References for SI reference citations

**
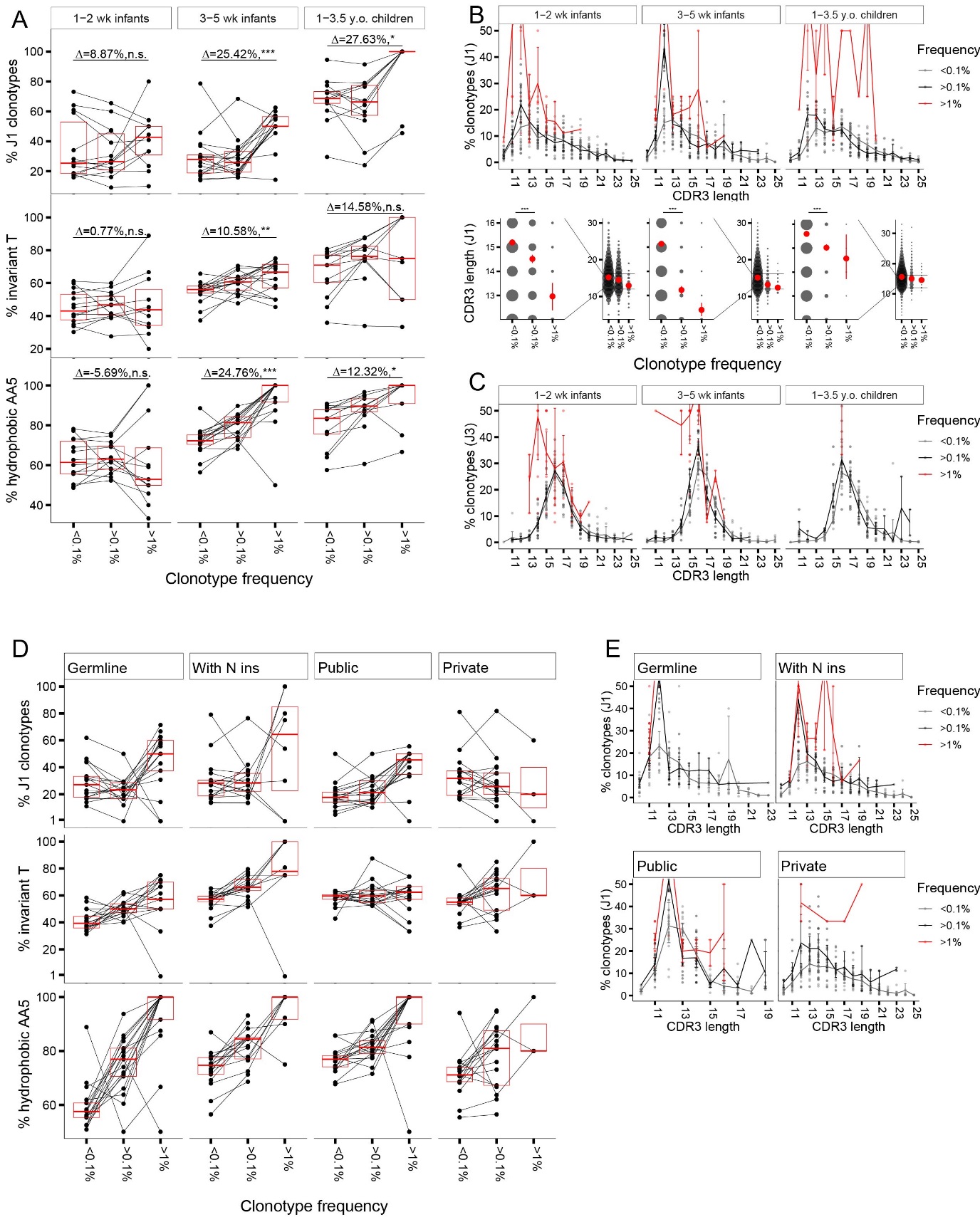
**

**Supplementary Figure 1.**

**A:** Proportion of sequences featuring the determinants of pAg reactivity in the infant and child repertoires (data retrieved from (1)): rearrangement to J1 region (top row), the invT (middle row) and a hAA5 (A/V/L/I/P/W/F/M)(bottom row) among clonotypes with increasing *f*. Lines connect individual donors, bars represent median values and IQRs. The median difference and the p-values were obtained using Wilcoxon rank-sum test. Note, in the repertoires of cells at 3-5 week of age, that in a large proportion of sequences the hAA5 is one of the non-invT encoded amino acids (A/P/W), whereas in the child and adult repertoires (see Fig1B-C) these are almost exclusively the invT-encoded ones. **B:** Top row: CDR3δ length distribution in the J1-rearranged sequences of the infant and child repertoires classified by clonotype frequency. Bottom row: corresponding summary graphs showing mean values and error bars representing 95% CI for the mean in red. *P < 0.05; **P ≤ 0.01; ***P ≤ 0.001 (Wilcoxon rank-sum test). **C:** CDR3δ length distribution in the J3-rearranged sequences of the infant and child repertoires classified by clonotype frequency. **D:** Analysis of the infant repertoires of 3-5 weeks of age, performed as in (A), after separating subrepertoires generated without (germline) and with N additions, as well as separate analysis of pubic (sequences shared between donors within the study(1)) versus private sequences. Lines connect individual donors, bars represent median values and IQRs. **E:** CDR3δ length distribution in the J1-rearranged sequences of the infant repertoires of 3-5 weeks of age, after separating subrepertoires generated without and with N additions, as well as separate analysis of pubic versus private sequences.


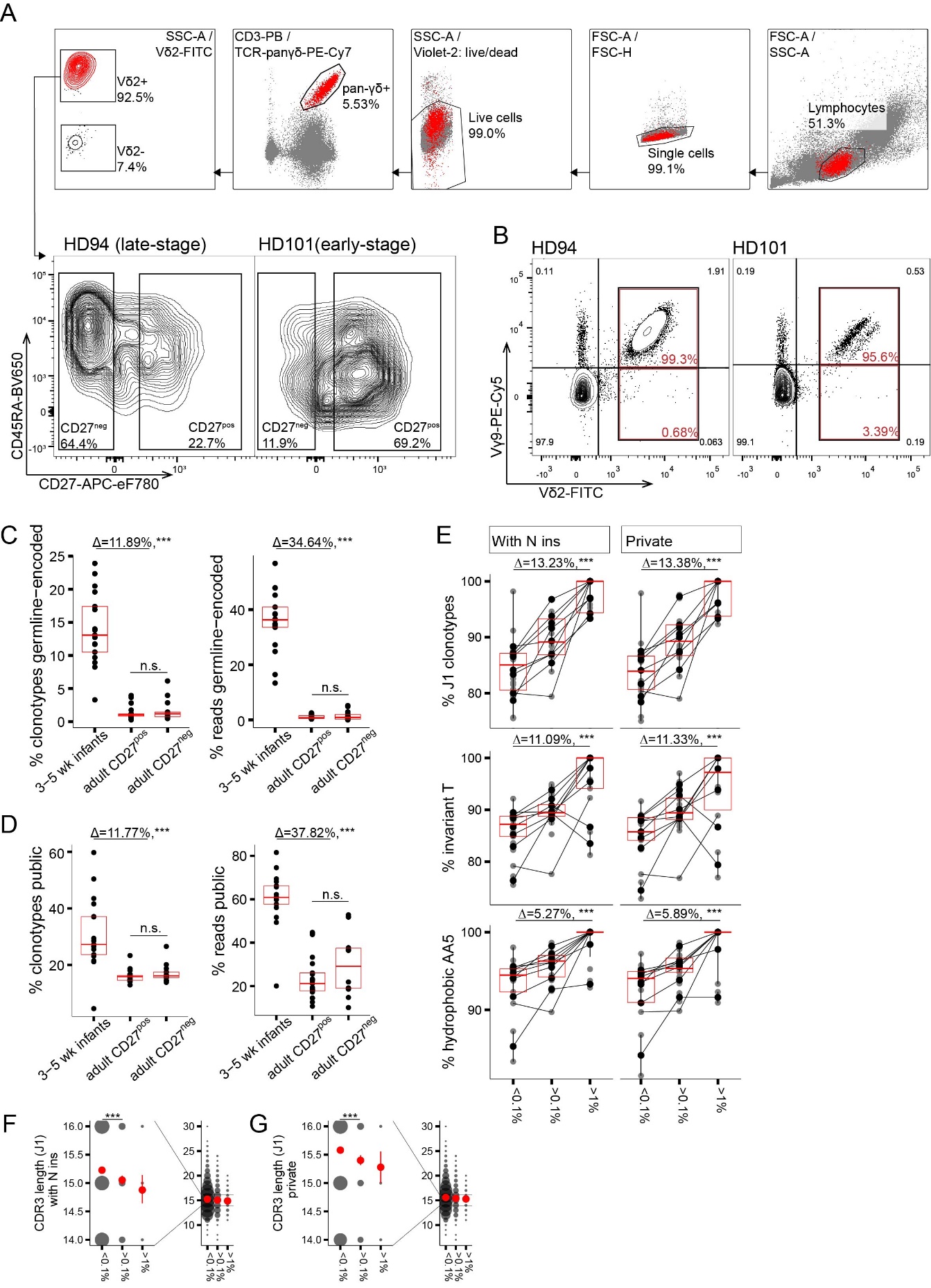


**Supplementary Figure 2.**

**A:** Vδ2+ cell phenotype and sorting strategy on the examples of donors 94 (late-stage profile) and 101 (early-stage profile). **B:** Expression of the Vγ9 chain on the Vδ2pos cells within the sorting gate. **C:** Percentage of germline-encoded clonotypes (left graph), as well as repertoire space occupied by germline-encoded clonotypes (right graph), in the repertoires of infants at 3-5 weeks of age versus adult repertoires. Bars represent median values and IQRs. **D:** Percentage of public clonotypes (left graph), as well as repertoire space occupied by public clonotypes (right graph), in the repertoires of infants at 3-5 weeks of age versus adult repertoires. Bars represent median values and IQRs. **E:** Enrichment in the pAg-reactivity determinants in the repertoires of CD27^pos^ cells of adults shown as in main Figure 1A-C, after the germline-encoded (left column) or the public (right column) AA sequences were excluded. Lines connect individual donors; bars represent median values and IQRs. The median difference and the p-values were obtained using Wilcoxon rank-sum test. **F-G:** CDR3δ length distribution of the J1-rearranged repertoires plotted against clonotype frequency in the CD27^pos^ cells of adults after filtering out germline-encoded (F) or public (G) sequences. Mean values and error bars representing 95% CI for the mean are shown in red. *P < 0.05; **P ≤ 0.01; ***P ≤ 0.001.


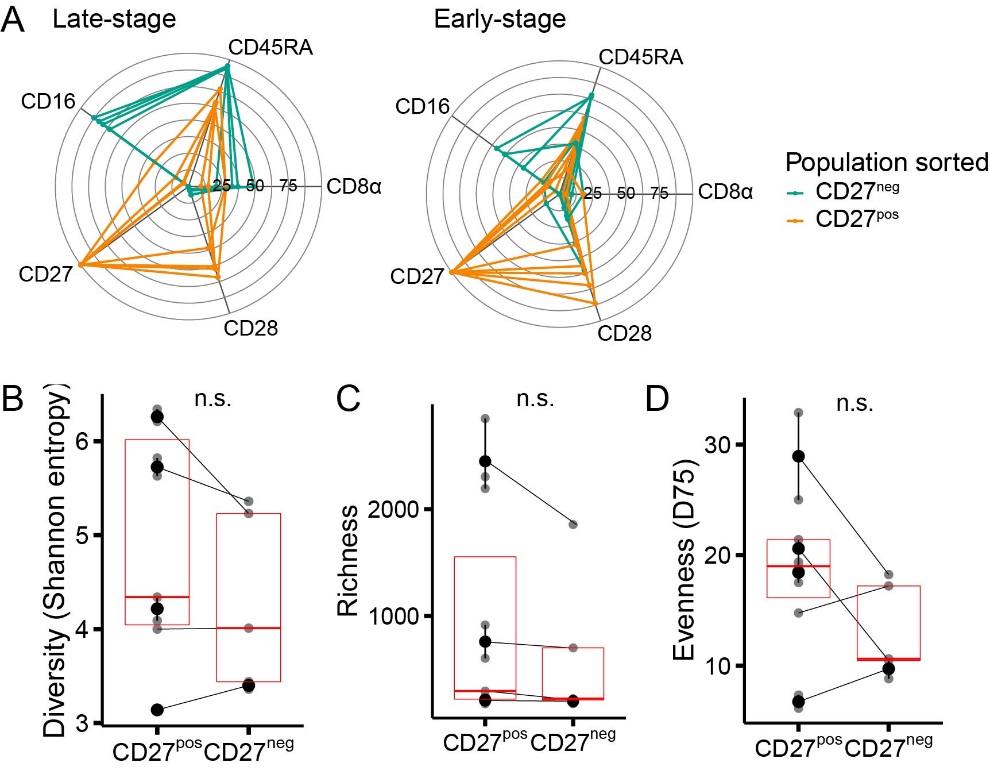


**Supplementary Figure 3.**

**A:** Phenotype of the sorted populations in the late-stage (n=4) and the early-stage (n=5) phenotype donors. Percentage of cells positive for a given marker within the sorted subsets is indicated, lines are connecting data within a donor. **B-D:** Measures of diversity in the early-stage profile donors: Shannon entropy (B), repertoire richness (C), repertoire evenness (D). Lines connect the average of the replicates in each donor. Bars represent median values and interquartile ranges (IQRs), the p-values were determined using Wilcoxon rank-sum test. *P < 0.05; **P ≤ 0.01; ***P ≤ 0.001.

**
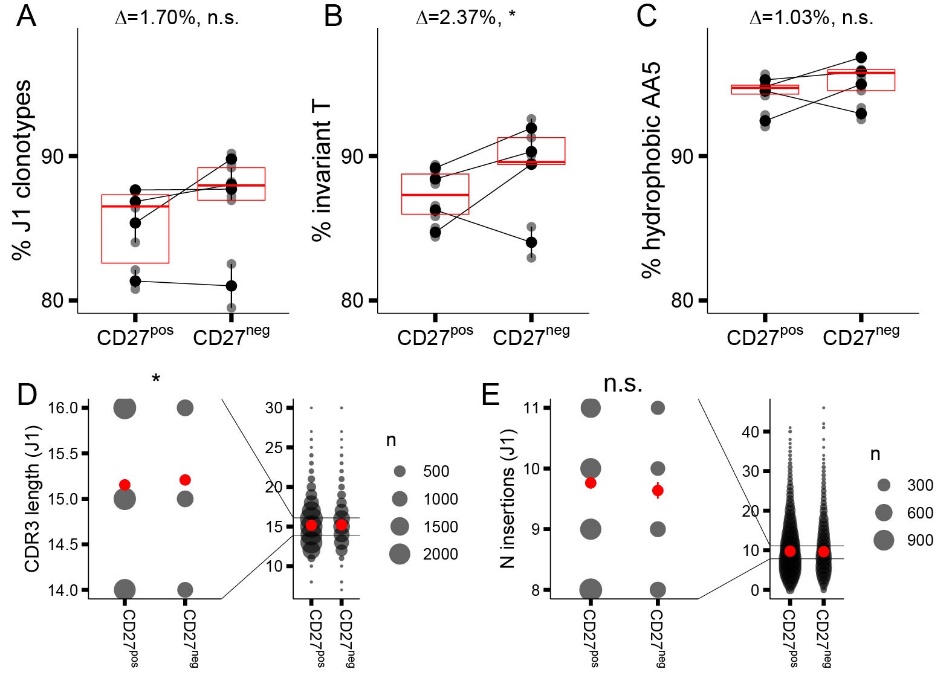
**

**Supplementary Figure 4.**

**A-C:** Comparison between complete repertoires of the sorted CD27^pos^ and CD27^neg^ populations of the late-stage donors: proportion of sequences rearranged to J1 region (A), proportion of sequences featuring the invT (B), proportion of sequences featuring hAA5 (C). Lines connect the average of the replicates in each donor. Bars represent medians and IQRs. The median difference and the p-value were obtained using Wilcoxon rank-sum test. **D-E:** CDR3δ length (D) and number of N insertions (E) in clonotypes rearranged to J1 region. Mean values and error bars representing 95% CI for the mean are shown in red (Wilcoxon rank-sum test). *P < 0.05; n.s. not significant.

**
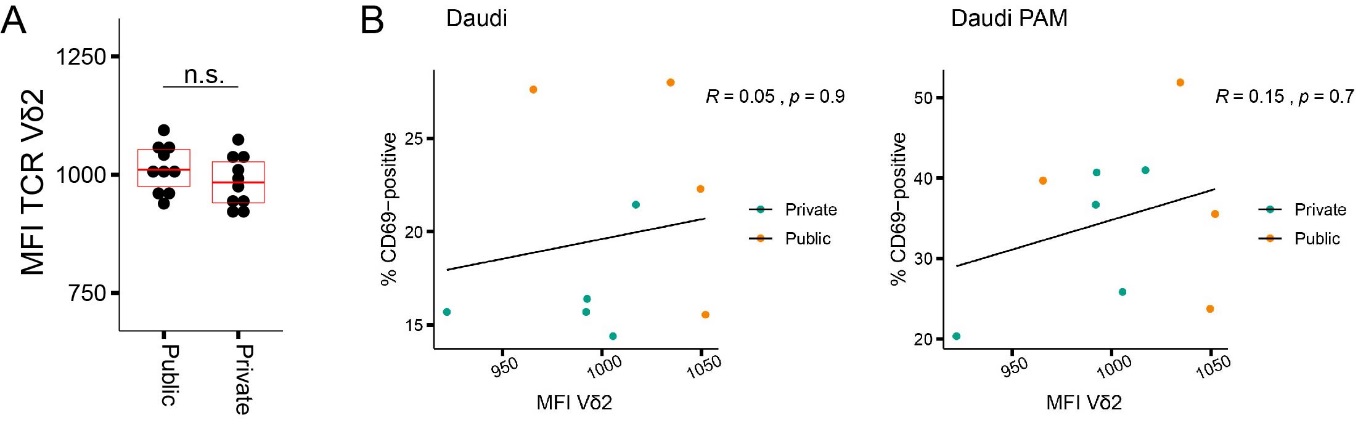
**

**Supplementary Figure 5.**

**A:** TCR expression level of the Jurkat76 transductants. **B:** TCR expression level of the Jurkat76 transductants plotted against CD69 upregulation level, showing no significant correlation (Spearman’s ρ).

**
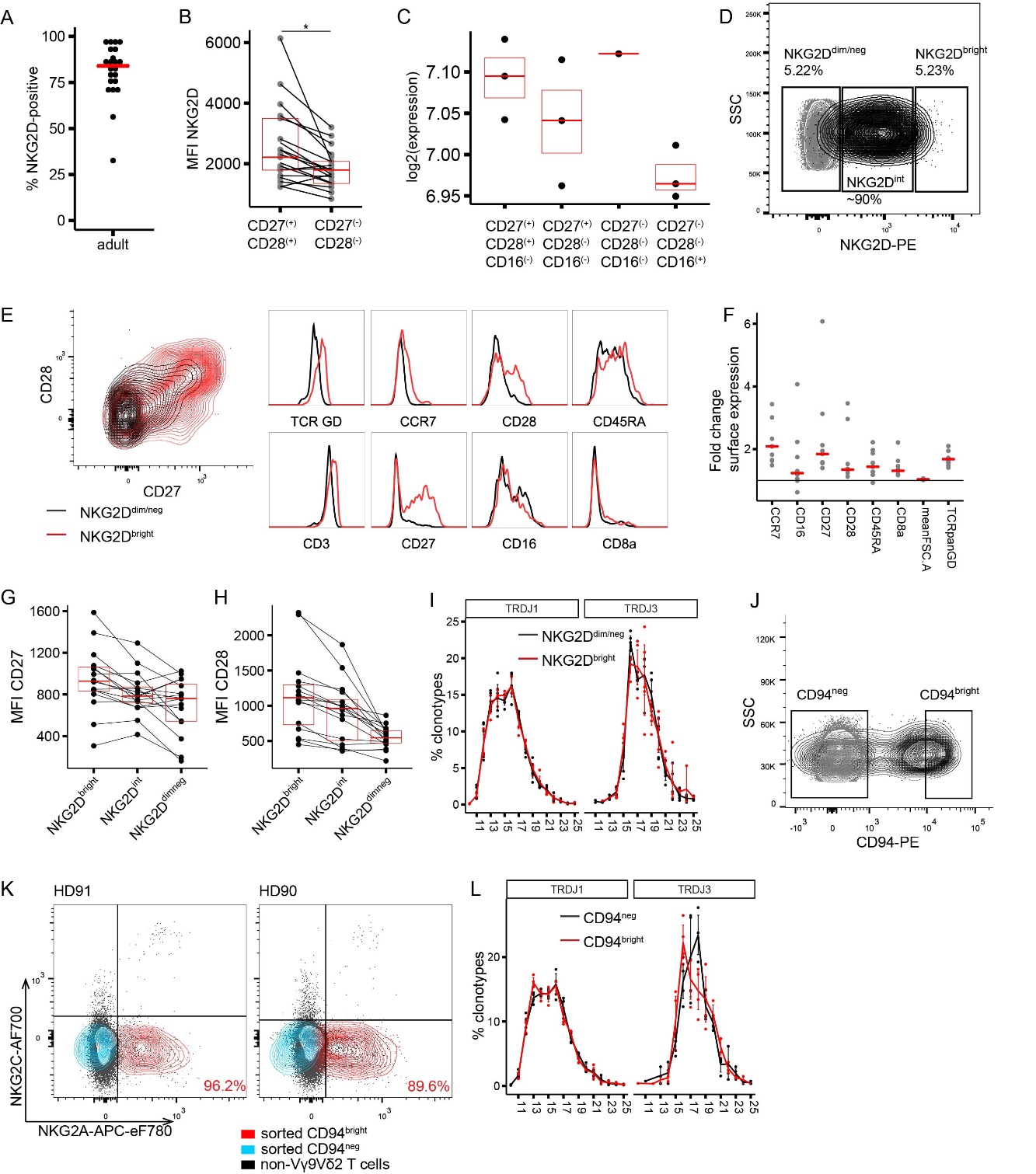
Supplementary Figure 6.**

**A:** Percentage NKG2D-positive Vδ2+ T cells in the adult donors(n=22). Bar represents the median value. **B:** MFI NKG2D expression on the cells of the CD27^pos^CD28^pos^ versus CD27^neg^CD28^neg^ phenotype (n=19 donors). Lines connect populations which belong to one donor, bars represent median values. P-value was determined using Wilcoxon rank-sum test. **C:** NKG2D mRNA expression level in the dataset published by Ryan et al.(2). **D:** NKG2D expression on the Vδ2+ population (in black) and identification of the NKG2D^dim/neg^ and NKG2D^bright^ cells. CD4+ αβT cells in the same donor (gated on CD3^pos^ CD4^pos^ pan-γδTCR^neg^, light gray color) were used for setting the gate for NKG2D^dim/neg^ cells. **E:** Example of HD94 (late-stage profile donor): expression of the differentiation markers CD27 and CD28, CCR7, CD45RA, CD16, TCR and CD8α on the NKG2D^bright^ vs NKG2D^dim/neg^ cells. **F:** Expression of the differentiation markers, TCRγδ, as well as CD8α on the NKG2D^bright^ vs NKG2D^dim/neg^ cells in 9 out of 12 donors with concordant phenotypes, as demonstrated in the Figure S5B. **G-H:** Expression level of CD27(G) and CD28(H) on the subpopulations gated as in D. **I:** CDR3δ length distribution among clonotypes rearranged to J1 and J3 region in the NKG2D^bright^ vs NKG2D^dim/neg^ cells. **J:** CD94 expression on the Vδ2+ population (in black) and the sorting strategy. Again, the CD4+ αβT cells in the same donor were used to gate for CD94^neg^ cells. **K:** Expression of NKG2A(B) versus NKG2C on the sorted CD94^bright^ (red) and CD94^neg^ (blue) cells (percentage NKG2A-positive cells of the sorted CD94^bright^ population is indicated). Non-Vγ9Vδ2 cells are shown in grey for comparison. **L:** CDR3δ length distribution among clonotypes rearranged to J1 and J3 region in the CD94^bright^ and CD94^neg^ populations sorted.

**Supplementary Table 1.** Public AA clonotypes shared among >50% donors in this study and corresponding public and private nucleotypes. Number of donors in which the sequence occurs in this study, as well as occurrence in other published repertoire studies are indicated. Nucleotides are annotated using IMGT Junction Analysis tool(3): V and J gene in light grey, D gene in dark grey, N insertions in black, while an underscore denotes P nucleotides.

| Nucleotypes | Public CDR3δ amino acid clonotypes and  corresponding nucleotypes | | | | | | | | | | | | | | | | | | CDR3δ length | N insertions | | | Janssen et al, 2020 | Ravens et al, 2017 | Davey et al, 2017 | Ravens et al, 2020 | Papadopoulou et al, 2020 |
| --- | --- | --- | --- | --- | --- | --- | --- | --- | --- | --- | --- | --- | --- | --- | --- | --- | --- | --- | --- | --- | --- | --- | --- | --- | --- | --- | --- |
|  |  |  |  |  |  |  |  |  |  |  |  |  |  |  |  |  |  |  |  | VD | DJ | Total |  |  |  |  |  |
|  | **C** | **A** | **C** | **D** | **T** | **L** | **G** | **D** | **T** | **D** | **K** | **L** | **I** | **F** |  | |  |  | 12 |  |  |  | **+** | **+** | **+** | **+** | **+** |
| Public | *TGT* | *GCC* | *TGT* | *GAC* | *ACC* | *CTG* | *GGG* | *GAT* | *ACC* | *GAT* | *AAA* | *CTC* | *ATC* | *TTT* |  |  |  |  |  | 0 | 0 | 0 |  |  |  |  |  |
|  | *TGT* | *GCC* | *TGT* | *GAC* | *ACT* | *CTG* | *GGG* | *GAC* | *ACC* | *GAT* | *AAA* | *CTC* | *ATC* | *TTT* |  |  |  |  |  | 1 | 0 | 1 |  |  |  |  |  |
|  | *TGT* | *GCC* | *TGT* | *GAC* | *ACC* | *CTG* | *GGG* | *GAC* | *ACC* | *GAT* | *AAA* | *CTC* | *ATC* | *TTT* |  |  |  |  |  | 0 | 0 | 0 |  |  |  |  |  |
|  | *TGT* | *GCC* | *TGT* | *GAC* | *ACG* | *CTG* | *GGG* | *GAC* | *ACC* | *GAT* | *AAA* | *CTC* | *ATC* | *TTT* |  |  |  |  |  | 1 | 0 | 1 |  |  |  |  |  |
|  | *TGT* | *GCC* | *TGT* | *GAC* | *ACA* | *CTG* | *GGG* | *GAC* | *ACC* | *GAT* | *AAA* | *CTC* | *ATC* | *TTT* |  |  |  |  |  | 0 | 0 | 0 |  |  |  |  |  |
|  | *TGT* | *GCC* | *TGT* | *GAC* | *ACG* | *CTG* | *GGG* | *GAT* | *ACC* | *GAT* | *AAA* | *CTC* | *ATC* | *TTT* |  |  |  |  |  | 1 | 0 | 1 |  |  |  |  |  |
| Private | TGT | GCC | TGT | GAC | ACA | CTG | GGG | GAT | ACC | GAT | AAA | CTC | ATC | TTT |  |  |  |  |  | 0 | 0 | 0 |  |  |  |  |  |
|  | TGT | GCC | TGT | GAC | ACC | TTG | GGG | GAT | ACC | GAT | AAA | CTC | ATC | TTT |  |  |  |  |  | 1 | 0 | 1 |  |  |  |  |  |
|  | TGT | GCC | TGT | GAC | ACC | TTG | GGG | GAT | ACG | GAT | AAA | CTC | ATC | TTT |  |  |  |  |  | 1 | 0 | 1 |  |  |  |  |  |
|  | TGT | GCC | TGT | GAC | ACG | TTG | GGG | GAC | ACC | GAT | AAA | CTC | ATC | TTT |  |  |  |  |  | 2 | 0 | 2 |  |  |  |  |  |
|  | TGT | GCC | TGT | GAC | ACC | CTC | GGG | GAT | ACC | GAT | AAA | CTC | ATC | TTT |  |  |  |  |  | 3 | 0 | 3 |  |  |  |  |  |
|  | TGT | GCC | TGT | GAC | ACT | TTG | GGG | GAT | ACT | GAT | AAA | CTC | ATC | TTT |  |  |  |  |  | 2 | 1 | 3 |  |  |  |  |  |
|  | TGT | GCC | TGT | GAC | ACA | CTG | GGG | GAT | ACG | GAT | AAA | CTC | ATC | TTT |  |  |  |  |  | 0 | 0 | 0 |  |  |  |  |  |
|  | TGT | GCC | TGT | GAC | ACA | CTG | GGC | GAC | ACC | GAT | AAA | CTC | ATC | TTT |  |  |  |  |  | 0 | 2 | 2 |  |  |  |  |  |
|  | TGT | GCC | TGT | GAC | ACG | CTA | GGG | GAT | ACC | GAT | AAA | CTC | ATC | TTT |  |  |  |  |  | 4 | 0 | 4 |  |  |  |  |  |
|  | TGT | GCC | TGT | GAC | ACG | CTG | GGG | GAT | ACG | GAT | AAA | CTC | ATC | TTT |  |  |  |  |  | 1 | 0 | 1 |  |  |  |  |  |
|  | TGT | GCC | TGT | GAT | ACT | CTG | GGG | GAT | ACC | GAT | AAA | CTC | ATC | TTT |  |  |  |  |  | 4 | 0 | 4 |  |  |  |  |  |
|  | TGT | GCC | TGT | GAC | ACT | CTG | GGG | GAT | ACC | GAT | AAA | CTC | ATC | TTT |  |  |  |  |  | 1 | 0 | 1 |  |  |  |  |  |
|  | TGT | GCC | TGT | GAC | ACG | TTG | GGG | GAT | ACC | GAT | AAA | CTC | ATC | TTT |  |  |  |  |  | 2 | 0 | 2 |  |  |  |  |  |
|  | **C** | **A** | **C** | **D** | **T** | **L** | **L** | **G** | **D** | **T** | **D** | **K** | **L** | **I** | **F** |  |  |  | 13 |  |  |  | **-** | **+** | **+** | **-** | **-** |
| Public | *TGT* | *GCC* | *TGT* | *GAC* | *ACG* | *TTA* | *CTG* | *GGG* | *GAC* | *ACC* | *GAT* | *AAA* | *CTC* | *ATC* | *TTT* |  |  |  |  | 2 | 0 | 2 |  |  |  |  |  |
|  | *TGT* | *GCC* | *TGT* | *GAC* | *ACG* | *TTA* | *CTG* | *GGG* | *GAT* | *ACC* | *GAT* | *AAA* | *CTC* | *ATC* | *TTT* |  |  |  |  | 2 | 0 | 2 |  |  |  |  |  |
|  | *TGT* | *GCC* | *TGT* | *GAC* | *ACC* | *TTA* | *CTG* | *GGG* | *GAC* | *ACC* | *GAT* | *AAA* | *CTC* | *ATC* | *TTT* |  |  |  |  | 1 | 0 | 1 |  |  |  |  |  |
|  | *TGT* | *GCC* | *TGT* | *GAC* | *ACC* | *CTA* | *CTG* | *GGG* | *GAT* | *ACC* | *GAT* | *AAA* | *CTC* | *ATC* | *TTT* |  |  |  |  | 1 | 0 | 1 |  |  |  |  |  |
|  | *TGT* | *GCC* | *TGT* | *GAC* | *ACT* | *CTA* | *CTG* | *GGG* | *GAT* | *ACC* | *GAT* | *AAA* | *CTC* | *ATC* | *TTT* |  |  |  |  | 2 | 0 | 2 |  |  |  |  |  |
| Private | TGT | GCC | TGT | GAC | ACC | TTG | CTG | GGG | GAC | ACC | GAT | AAA | CTC | ATC | TTT |  |  |  |  | 3 | 0 | 3 |  |  |  |  |  |
|  | TGT | GCC | TGT | GAC | ACC | CTA | CTG | GGG | GAT | ACG | GAT | AAA | CTC | ATC | TTT |  |  |  |  | 1 | 0 | 1 |  |  |  |  |  |
|  | TGT | GCC | TGT | GAC | ACC | TTG | CTG | GGG | GAT | ACC | GAT | AAA | CTC | ATC | TTT |  |  |  |  | 3 | 0 | 3 |  |  |  |  |  |
|  | TGT | GCC | TGT | GAC | ACT | TTA | CTG | GGG | GAC | ACC | GAT | AAA | CTC | ATC | TTT |  |  |  |  | 2 | 0 | 2 |  |  |  |  |  |
|  | TGT | GCC | TGT | GAC | ACC | TTA | CTG | GGG | GAT | ACC | GAT | AAA | CTC | ATC | TTT |  |  |  |  | 1 | 0 | 1 |  |  |  |  |  |
|  | TGT | GCC | TGT | GAT | ACC | TTA | CTG | GGG | GAT | ACC | GAT | AAA | CTC | ATC | TTT |  |  |  |  | 2 | 0 | 2 |  |  |  |  |  |
|  | TGT | GCC | TGT | GAC | ACT | TTA | CTG | GGG | GAT | ACG | GAT | AAA | CTC | ATC | TTT |  |  |  |  | 2 | 0 | 2 |  |  |  |  |  |
|  | TGT | GCC | TGT | GAC | ACG | CTA | CTG | GGG | GAC | ACC | GAT | AAA | CTC | ATC | TTT |  |  |  |  | 2 | 0 | 2 |  |  |  |  |  |
|  | TGT | GCC | TGT | GAC | ACC | CTC | CTG | GGG | GAC | ACC | GAT | AAA | CTC | ATC | TTT |  |  |  |  | 3 | 0 | 3 |  |  |  |  |  |
|  | **C** | **A** | **C** | **D** | **V** | **L** | **G** | **D** | **T** | **D** | **K** | **L** | **I** | **F** |  | |  |  | 12 |  |  |  | **+** | **+** | **+** | **+** | **+** |
| Pub | *TGT* | *GCC* | *TGT* | *GAC* | *GTA* | *CTG* | *GGG* | *GAC* | *ACC* | *GAT* | *AAA* | *CTC* | *ATC* | *TTT* |  |  |  |  |  | 0 | 0 | 0 |  |  |  |  |  |
|  | *TGT* | *GCC* | *TGT* | *GAC* | *GTA* | *CTG* | *GGG* | *GAT* | *ACC* | *GAT* | *AAA* | *CTC* | *ATC* | *TTT* |  |  |  |  |  | 0 | 0 | 0 |  |  |  |  |  |
| Private | TGT | GCC | TGT | GAC | GTA | CTG | GGG | GAT | ACG | GAT | AAA | CTC | ATC | TTT |  |  |  |  |  | 0 | 0 | 0 |  |  |  |  |  |
|  | TGT | GCC | TGT | GAC | GTC | CTG | GGG | GAC | ACC | GAT | AAA | CTC | ATC | TTT |  |  |  |  |  | 3 | 0 | 3 |  |  |  |  |  |
|  | TGT | GCC | TGT | GAT | GTC | CTG | GGG | GAC | ACC | GAT | AAA | CTC | ATC | TTT |  |  |  |  |  | 4 | 0 | 4 |  |  |  |  |  |
|  | TGT | GCC | TGT | GAT | GTA | CTG | GGG | GAT | ACC | GAT | AAA | CTC | ATC | TTT |  |  |  |  |  | 1 | 0 | 1 |  |  |  |  |  |
|  | **C** | **A** | **C** | **D** | **T** | **L** | **G** | **V** | **Y** | **T** | **D** | **K** | **L** | **I** | **F** |  |  |  | 13 |  |  |  | **+** | **-** | **-** | **-** | **-** |
| P | *TGT* | *GCC* | *TGT* | *GAC* | *ACC* | *CTG* | *GGG* | *GTT* | *TAC* | *ACC* | *GAT* | *AAA* | *CTC* | *ATC* | *TTT* |  |  |  |  | 0 | 2 | 2 |  |  |  |  |  |
| Private | TGT | GCC | TGT | GAC | ACA | CTG | GGG | GTC | TAC | ACC | GAT | AAA | CTC | ATC | TTT |  |  |  |  | 0 | 2 | 2 |  |  |  |  |  |
|  | TGT | GCC | TGT | GAC | ACC | TTG | GGG | GTT | TAC | ACC | GAT | AAA | CTC | ATC | TTT |  |  |  |  | 1 | 2 | 3 |  |  |  |  |  |
|  | TGT | GCC | TGT | GAC | ACT | CTG | GGG | GTT | TAC | ACC | GAT | AAA | CTC | ATC | TTT |  |  |  |  | 1 | 2 | 3 |  |  |  |  |  |
|  | TGT | GCC | TGT | GAC | ACC | CTG | GGG | GTG | TAC | ACC | GAT | AAA | CTC | ATC | TTT |  |  |  |  | 0 | 0 | 0 |  |  |  |  |  |
|  | TGT | GCC | TGT | GAC | ACG | CTG | GGG | GTC | TAC | ACC | GAT | AAA | CTC | ATC | TTT |  |  |  |  | 1 | 2 | 3 |  |  |  |  |  |
|  | TGT | GCC | TGT | GAC | ACC | CTG | GGG | GTC | TAC | ACC | GAT | AAA | CTC | ATC | TTT |  |  |  |  | 0 | 2 | 2 |  |  |  |  |  |
|  | TGT | GCC | TGT | GAC | ACT | TTG | GGG | GTG | TAC | ACC | GAT | AAA | CTC | ATC | TTT |  |  |  |  | 2 | 0 | 2 |  |  |  |  |  |
|  | TGT | GCC | TGT | GAC | ACA | CTG | GGA | GTT | TAC | ACC | GAT | AAA | CTC | ATC | TTT |  |  |  |  | 0 | 4 | 4 |  |  |  |  |  |
|  | **C** | **A** | **C** | **D** | **T** | **V** | **G** | **D** | **T** | **D** | **K** | **L** | **I** | **F** |  | |  |  | 12 |  |  |  | **+** | **+** | **+** | **+** | **+** |
| Pu | *TGT* | *GCC* | *TGT* | *GAC* | *ACC* | *GTG* | *GGG* | *GAT* | *ACC* | *GAT* | *AAA* | *CTC* | *ATC* | *TTT* |  |  |  |  |  | 0 | 0 | 0 |  |  |  |  |  |
|  | *TGT* | *GCC* | *TGT* | *GAC* | *ACC* | *GTG* | *GGG* | *GAT* | *ACG* | *GAT* | *AAA* | *CTC* | *ATC* | *TTT* |  |  |  |  |  | 0 | 0 | 0 |  |  |  |  |  |
| Private | TGT | GCC | TGT | GAC | ACC | GTA | GGA | GAC | ACC | GAT | AAA | CTC | ATC | TTT |  |  |  |  |  | - | - | 6 |  |  |  |  |  |
|  | TGT | GCC | TGT | GAC | ACC | GTG | GGG | GAC | ACC | GAT | AAA | CTC | ATC | TTT |  |  |  |  |  | 0 | 0 | 0 |  |  |  |  |  |
|  | TGT | GCC | TGT | GAC | ACT | GTG | GGG | GAT | ACC | GAT | AAA | CTC | ATC | TTT |  |  |  |  |  | 2 | 0 | 2 |  |  |  |  |  |
|  | TGT | GCC | TGT | GAC | ACC | GTG | GGG | GAT | ACT | GAT | AAA | CTC | ATC | TTT |  |  |  |  |  | 0 | 1 | 1 |  |  |  |  |  |
|  | TGT | GCC | TGT | GAC | ACC | GTC | GGA | GAC | ACC | GAT | AAA | CTC | ATC | TTT |  |  |  |  |  | - | - | 6 |  |  |  |  |  |
|  | TGT | GCC | TGT | GAC | ACC | GTC | GGG | GAT | ACC | GAT | AAA | CTC | ATC | TTT |  |  |  |  |  | 2 | 0 | 2 |  |  |  |  |  |
|  | TGT | GCC | TGT | GAC | ACG | GTG | GGG | GAT | ACC | GAT | AAA | CTC | ATC | TTT |  |  |  |  |  | 2 | 0 | 2 |  |  |  |  |  |
|  | TGT | GCC | TGT | GAC | ACC | GTA | GGG | GAT | ACC | GAT | AAA | CTC | ATC | TTT |  |  |  |  |  | 2 | 0 | 2 |  |  |  |  |  |
|  | **C** | **A** | **C** | **D** | **T** | **V** | **G** | **E** | **Y** | **T** | **D** | **K** | **L** | **I** | **F** |  |  |  | 13 |  |  |  | **+** | **+** | **+** | **-** | **-** |
| Pu | *TGT* | *GCC* | *TGT* | *GAC* | *ACC* | *GTG* | *GGG* | *GAG* | *TAC* | *ACC* | *GAT* | *AAA* | *CTC* | *ATC* | *TTT* |  |  |  |  | 0 | 0 | 0 |  |  |  |  |  |
|  | *TGT* | *GCC* | *TGT* | *GAC* | *ACC* | *GTT* | *GGG* | *GAA* | *TAC* | *ACC* | *GAT* | *AAA* | *CTC* | *ATC* | *TTT* |  |  |  |  | 2 | 1 | 3 |  |  |  |  |  |
| Private | TGT | GCC | TGT | GAC | ACG | GTG | GGG | GAG | TAC | ACC | GAT | AAA | CTC | ATC | TTT |  |  |  |  | 2 | 0 | 2 |  |  |  |  |  |
|  | TGT | GCC | TGT | GAC | ACC | GTC | GGG | GAA | TAC | ACC | GAT | AAA | CTC | ATC | TTT |  |  |  |  | 2 | 1 | 3 |  |  |  |  |  |
|  | TGT | GCC | TGT | GAC | ACC | GTG | GGT | GAG | TAC | ACC | GAT | AAA | CTC | ATC | TTT |  |  |  |  | 0 | 3 | 3 |  |  |  |  |  |
|  | TGT | GCC | TGT | GAC | ACC | GTA | GGG | GAA | TAC | ACC | GAT | AAA | CTC | ATC | TTT |  |  |  |  | 2 | 1 | 3 |  |  |  |  |  |
|  | TGT | GCC | TGT | GAC | ACC | GTA | GGG | GAG | TAC | ACC | GAT | AAA | CTC | ATC | TTT |  |  |  |  | 2 | 0 | 2 |  |  |  |  |  |
|  | **C** | **A** | **C** | **D** | **T** | **V** | **G** | **G** | **Y** | **T** | **D** | **K** | **L** | **I** | **F** |  |  |  | 13 |  |  |  | **-** | **+** | **+** | **-** | **-** |
| Public | *TGT* | *GCC* | *TGT* | *GAC* | *ACC* | *GTT* | *GGG* | *GGA* | *TAC* | *ACC* | *GAT* | *AAA* | *CTC* | *ATC* | *TTT* |  |  |  |  | 1 | 0 | 1 |  |  |  |  |  |
|  | *TGT* | *GCC* | *TGT* | *GAC* | *ACC* | *GTC* | *GGG* | *GGA* | *TAC* | *ACC* | *GAT* | *AAA* | *CTC* | *ATC* | *TTT* |  |  |  |  | 2 | 0 | 2 |  |  |  |  |  |
|  | *TGT* | *GCC* | *TGT* | *GAC* | *ACC* | *GTG* | *GGG* | *GGG* | *TAC* | *ACC* | *GAT* | *AAA* | *CTC* | *ATC* | *TTT* |  |  |  |  | 0 | 1 | 1 |  |  |  |  |  |
| Private | TGT | GCC | TGT | GAC | ACC | GTC | GGG | GGG | TAC | ACC | GAT | AAA | CTC | ATC | TTT |  |  |  |  | 3 | 0 | 3 |  |  |  |  |  |
|  | TGT | GCC | TGT | GAC | ACC | GTT | GGG | GGT | TAC | ACC | GAT | AAA | CTC | ATC | TTT |  |  |  |  | 1 | 1 | 2 |  |  |  |  |  |
|  | TGT | GCC | TGT | GAC | ACG | GTG | GGG | GGA | TAC | ACC | GAT | AAA | CTC | ATC | TTT |  |  |  |  | 4 | 0 | 4 |  |  |  |  |  |
|  | TGT | GCC | TGT | GAC | ACC | GTC | GGG | GGC | TAC | ACC | GAT | AAA | CTC | ATC | TTT |  |  |  |  | 2 | 1 | 3 |  |  |  |  |  |
|  | TGT | GCC | TGT | GAC | ACG | GTG | GGG | GGG | TAC | ACC | GAT | AAA | CTC | ATC | TTT |  |  |  |  | 2 | 1 | 3 |  |  |  |  |  |
|  | TGT | GCC | TGT | GAC | ACC | GTT | GGG | GGG | TAC | ACC | GAT | AAA | CTC | ATC | TTT |  |  |  |  | 1 | 0 | 1 |  |  |  |  |  |
|  | TGT | GCC | TGT | GAC | ACC | GTA | GGG | GGC | TAC | ACC | GAT | AAA | CTC | ATC | TTT |  |  |  |  | 2 | 1 | 3 |  |  |  |  |  |
|  | TGT | GCC | TGT | GAC | ACT | GTT | GGG | GGA | TAC | ACC | GAT | AAA | CTC | ATC | TTT |  |  |  |  | 3 | 0 | 3 |  |  |  |  |  |
|  | TGT | GCC | TGT | GAC | ACC | GTA | GGG | GGA | TAC | ACC | GAT | AAA | CTC | ATC | TTT |  |  |  |  | 2 | 0 | 2 |  |  |  |  |  |
|  | TGT | GCC | TGT | GAC | ACG | GTG | GGG | GGT | TAC | ACC | GAT | AAA | CTC | ATC | TTT |  |  |  |  | 2 | 2 | 4 |  |  |  |  |  |
|  | TGT | GCC | TGT | GAC | ACG | GTT | GGG | GGA | TAC | ACC | GAT | AAA | CTC | ATC | TTT |  |  |  |  | 3 | 0 | 3 |  |  |  |  |  |
|  | **C** | **A** | **C** | **D** | **I** | **L** | **G** | **D** | **T** | **D** | **K** | **L** | **I** | **F** |  |  |  |  | 12 |  |  |  | **+** | **+** | **+** | **+** | **+** |
| Pub | *TGT* | *GCC* | *TGT* | *GAC* | *ATA* | *CTG* | *GGG* | *GAC* | *ACC* | *GAT* | *AAA* | *CTC* | *ATC* | *TTT* |  |  |  |  |  | 0 | 0 | 0 |  |  |  |  |  |
|  | *TGT* | *GCC* | *TGT* | *GAC* | *ATA* | *CTG* | *GGG* | *GAT* | *ACC* | *GAT* | *AAA* | *CTC* | *ATC* | *TTT* |  |  |  |  |  | 0 | 0 | 0 |  |  |  |  |  |
| Privat | TGT | GCC | TGT | GAC | ATT | CTG | GGG | GAT | ACC | GAT | AAA | CTC | ATC | TTT |  |  |  |  |  | 2 | 0 | 2 |  |  |  |  |  |
|  | TGT | GCC | TGT | GAC | ATC | CTG | GGG | GAC | ACC | GAT | AAA | CTC | ATC | TTT |  |  |  |  |  | 2 | 0 | 2 |  |  |  |  |  |
|  | TGT | GCC | TGT | GAC | ATC | CTG | GGG | GAT | ACC | GAT | AAA | CTC | ATC | TTT |  |  |  |  |  | 2 | 0 | 2 |  |  |  |  |  |
|  | **C** | **A** | **C** | **D** | **P** | **L** | **G** | **D** | **T** | **D** | **K** | **L** | **I** | **F** |  |  |  |  | 12 |  |  |  | **+** | **-** | **+** | **-** | **-** |
| Pu | *TGT* | *GCC* | *TGT* | *GAC* | *CCC* | *CTG* | *GGG* | *GAC* | *ACC* | *GAT* | *AAA* | *CTC* | *ATC* | *TTT* |  |  |  |  |  | 3 | 0 | 3 |  |  |  |  |  |
|  | *TGT* | *GCC* | *TGT* | *GAC* | *CCA* | *CTG* | *GGG* | *GAC* | *ACC* | *GAT* | *AAA* | *CTC* | *ATC* | *TTT* |  |  |  |  |  | 2 | 0 | 2 |  |  |  |  |  |
| Private | TGT | GCC | TGT | GAT | CCT | CTG | GGG | GAC | ACC | GAT | AAA | CTC | ATC | TTT |  |  |  |  |  | 0 | 0 | 0 |  |  |  |  |  |
|  | TGT | GCC | TGT | GAC | CCA | CTG | GGG | GAT | ACG | GAT | AAA | CTC | ATC | TTT |  |  |  |  |  | 2 | 0 | 2 |  |  |  |  |  |
|  | TGT | GCC | TGT | GAC | CCA | CTG | GGG | GAT | ACC | GAT | AAA | CTC | ATC | TTT |  |  |  |  |  | 2 | 0 | 2 |  |  |  |  |  |
|  | TGT | GCC | TGT | GAC | CCT | CTG | GGG | GAT | ACC | GAT | AAA | CTC | ATC | TTT |  |  |  |  |  | 3 | 0 | 3 |  |  |  |  |  |
|  | TGT | GCC | TGT | GAC | CCC | CTT | GGA | GAC | ACC | GAT | AAA | CTC | ATC | TTT |  |  |  |  |  | 2 | 4 | 6 |  |  |  |  |  |
|  | TGT | GCC | TGT | GAT | CCA | CTG | GGG | GAC | ACC | GAT | AAA | CTC | ATC | TTT |  |  |  |  |  | 3 | 0 | 3 |  |  |  |  |  |
|  | TGT | GCC | TGT | GAC | CCT | TTG | GGG | GAT | ACC | GAT | AAA | CTC | ATC | TTT |  |  |  |  |  | 0 | 0 | 0 |  |  |  |  |  |
|  | TGT | GCC | TGT | GAC | CCA | CTG | GGA | GAC | ACC | GAT | AAA | CTC | ATC | TTT |  |  |  |  |  | 2 | 2 | 4 |  |  |  |  |  |
|  | TGT | GCC | TGT | GAC | CCC | CTC | GGC | GAC | ACC | GAT | AAA | CTC | ATC | TTT |  |  |  |  |  | 10 | 0 | 10 |  |  |  |  |  |
|  | TGT | GCC | TGT | GAC | CCT | CTA | GGG | GAC | ACC | GAT | AAA | CTC | ATC | TTT |  |  |  |  |  | 6 | 0 | 6 |  |  |  |  |  |
|  | **C** | **A** | **C** | **D** | **T** | **A** | **G** | **G** | **S** | **S** | **W** | **D** | **T** | **R** | **Q** | **M** | **F** | **F** | 16 |  |  |  | **+** | **-** | **+** | **-** | **-** |
| Pub | *TGT* | *GCC* | *TGT* | *GAC* | *ACC* | *GCG* | *GGG* | *GGG* | *AGC* | *TCC* | *TGG* | *GAC* | *ACC* | *CGA* | *CAG* | *ATG* | *TTT* | *TTC* |  | 3 | 0 | 3 |  |  |  |  |  |
|  | *TGT* | *GCC* | *TGT* | *GAC* | *ACC* | *GCT* | *GGG* | *GGA* | *AGC* | *TCC* | *TGG* | *GAC* | *ACC* | *CGA* | *CAG* | *ATG* | *TTT* | *TTC* |  | 0 | 0 | 0 |  |  |  |  |  |
| Private | TGT | GCC | TGT | GAC | ACC | GCG | GGG | GGA | AGC | TCC | TGG | GAC | ACC | CGA | CAG | ATG | TTT | TTC |  | 2 | 0 | 2 |  |  |  |  |  |
|  | TGT | GCC | TGT | GAC | ACT | GCT | GGG | GGT | AGC | TCC | TGG | GAC | ACC | CGA | CAG | ATG | TTT | TTC |  | 2 | 1 | 3 |  |  |  |  |  |
|  | TGT | GCC | TGT | GAC | ACC | GCT | GGG | GGG | AGC | TCC | TGG | GAC | ACC | CGA | CAG | ATG | TTT | TTC |  | 0 | 0 | 0 |  |  |  |  |  |
|  | TGT | GCC | TGT | GAC | ACA | GCT | GGG | GGA | AGC | TCC | TGG | GAC | ACC | CGA | CAG | ATG | TTT | TTC |  | 2 | 0 | 2 |  |  |  |  |  |
|  | **C** | **A** | **C** | **D** | **T** | **V** | **G** | **G** | **T** | **D** | **K** | **L** | **I** | **F** |  |  |  |  | 12 |  |  |  | **+** | **-** | **+** | **-** | **-** |
| Public | *TGT* | *GCC* | *TGT* | *GAC* | *ACC* | *GTC* | *GGG* | *GGG* | *ACC* | *GAT* | *AAA* | *CTC* | *ATC* | *TTT* |  |  |  |  |  | 3 | 0 | 3 |  |  |  |  |  |
|  | *TGT* | *GCC* | *TGT* | *GAC* | *ACC* | *GTT* | *GGG* | *GGT* | *ACC* | *GAT* | *AAA* | *CTC* | *ATC* | *TTT* |  |  |  |  |  | 1 | 1 | 2 |  |  |  |  |  |
|  | *TGT* | *GCC* | *TGT* | *GAC* | *ACC* | *GTG* | *GGG* | *GGT* | *ACC* | *GAT* | *AAA* | *CTC* | *ATC* | *TTT* |  |  |  |  |  | 0 | 2 | 2 |  |  |  |  |  |
| Private | TGT | GCC | TGT | GAC | ACC | GTT | GGC | GGC | ACC | GAT | AAA | CTC | ATC | TTT |  |  |  |  |  | - | - | 7 |  |  |  |  |  |
|  | TGT | GCC | TGT | GAC | ACC | GTT | GGG | GGC | ACC | GAT | AAA | CTC | ATC | TTT |  |  |  |  |  | 1 | 0 | 1 |  |  |  |  |  |
|  | TGT | GCC | TGT | GAC | ACC | GTG | GGG | GGC | ACC | GAT | AAA | CTC | ATC | TTT |  |  |  |  |  | 0 | 1 | 1 |  |  |  |  |  |
|  | TGT | GCC | TGT | GAC | ACC | GTA | GGT | GGG | ACC | GAT | AAA | CTC | ATC | TTT |  |  |  |  |  | 4 | 0 | 4 |  |  |  |  |  |
|  | TGT | GCC | TGT | GAC | ACC | GTA | GGC | GGA | ACC | GAT | AAA | CTC | ATC | TTT |  |  |  |  |  | - | - | 8 |  |  |  |  |  |
|  | TGT | GCC | TGT | GAC | ACC | GTC | GGT | GGA | ACC | GAT | AAA | CTC | ATC | TTT |  |  |  |  |  | - | - | 8 |  |  |  |  |  |
|  | **C** | **A** | **C** | **D** | **T** | **V** | **G** | **T** | **Y** | **T** | **D** | **K** | **L** | **I** | **F** |  |  |  | 13 |  |  |  | **+** | **+** | **+** | **-** | **-** |
| Private | TGT | GCC | TGT | GAC | ACT | GTG | GGG | ACG | TAC | ACC | GAT | AAA | CTC | ATC | TTT |  |  |  |  | 2 | 2 | 4 |  |  |  |  |  |
|  | TGT | GCC | TGT | GAC | ACC | GTC | GGG | ACA | TAC | ACC | GAT | AAA | CTC | ATC | TTT |  |  |  |  | 2 | 2 | 4 |  |  |  |  |  |
|  | TGT | GCC | TGT | GAC | ACT | GTG | GGG | ACT | TAC | ACC | GAT | AAA | CTC | ATC | TTT |  |  |  |  | 2 | 3 | 5 |  |  |  |  |  |
|  | TGT | GCC | TGT | GAC | ACC | GTG | GGG | ACC | TAC | ACC | GAT | AAA | CTC | ATC | TTT |  |  |  |  | 0 | 3 | 3 |  |  |  |  |  |
|  | TGT | GCC | TGT | GAC | ACC | GTG | GGG | ACT | TAC | ACC | GAT | AAA | CTC | ATC | TTT |  |  |  |  | 0 | 3 | 3 |  |  |  |  |  |
|  | TGT | GCC | TGT | GAC | ACC | GTG | GGA | ACA | TAC | ACC | GAT | AAA | CTC | ATC | TTT |  |  |  |  | 0 | 4 | 4 |  |  |  |  |  |
|  | TGT | GCC | TGT | GAC | ACC | GTA | GGG | ACT | TAC | ACC | GAT | AAA | CTC | ATC | TTT |  |  |  |  | 2 | 2 | 4 |  |  |  |  |  |
|  | TGT | GCC | TGT | GAC | ACC | GTG | GGG | ACA | TAC | ACC | GAT | AAA | CTC | ATC | TTT |  |  |  |  | 0 | 3 | 3 |  |  |  |  |  |
|  | **C** | **A** | **C** | **D** | **T** | **W** | **G** | **T** | **D** | **K** | **L** | **I** | **F** |  |  |  |  |  | 11 |  |  |  | **+** | **+** | **+** | **+** | **+** |
| P | *TGT* | *GCC* | *TGT* | *GAC* | *ACC* | *TGG* | *GGG* | *ACC* | *GAT* | *AAA* | *CTC* | *ATC* | *TTT* |  |  |  |  |  |  | 0 | 0 | 0 |  |  |  |  |  |
| Private | TGT | GCC | TGT | GAC | ACT | TGG | GGA | ACC | GAT | AAA | CTC | ATC | TTT |  |  |  |  |  |  | 2 | 0 | 2 |  |  |  |  |  |
|  | TGT | GCC | TGT | GAC | ACC | TGG | GGT | ACC | GAT | AAA | CTC | ATC | TTT |  |  |  |  |  |  | 0 | 1 | 1 |  |  |  |  |  |
|  | TGT | GCC | TGT | GAC | ACG | TGG | GGA | ACC | GAT | AAA | CTC | ATC | TTT |  |  |  |  |  |  | 1 | 1 | 2 |  |  |  |  |  |
|  | TGT | GCC | TGT | GAC | ACT | TGG | GGC | ACC | GAT | AAA | CTC | ATC | TTT |  |  |  |  |  |  | 1 | 0 | 1 |  |  |  |  |  |
|  | TGT | GCC | TGT | GAC | ACC | TGG | GGA | ACC | GAT | AAA | CTC | ATC | TTT |  |  |  |  |  |  | 0 | 1 | 1 |  |  |  |  |  |
|  | TGT | GCC | TGT | GAC | ACT | TGG | GGG | ACC | GAT | AAA | CTC | ATC | TTT |  |  |  |  |  |  | 1 | 0 | 1 |  |  |  |  |  |
|  | **C** | **A** | **C** | **D** | **T** | **W** | **G** | **Y** | **T** | **D** | **K** | **L** | **I** | **F** |  | |  |  | 12 |  |  |  | **+** | **+** | **+** | **-** | **+** |
| Pu | *TGT* | *GCC* | *TGT* | *GAC* | *ACC* | *TGG* | *GGG* | *TAC* | *ACC* | *GAT* | *AAA* | *CTC* | *ATC* | *TTT* |  |  |  |  |  | 0 | 0 | 0 |  |  |  |  |  |
|  | *TGT* | *GCC* | *TGT* | *GAC* | *ACC* | *TGG* | *GGA* | *TAC* | *ACC* | *GAT* | *AAA* | *CTC* | *ATC* | *TTT* |  |  |  |  |  | 1 | 0 | 1 |  |  |  |  |  |
| Privat | TGT | GCC | TGT | GAC | ACT | TGG | GGA | TAC | ACC | GAT | AAA | CTC | ATC | TTT |  |  |  |  |  | 2 | 0 | 2 |  |  |  |  |  |
|  | TGT | GCC | TGT | GAC | ACC | TGG | GGC | TAC | ACC | GAT | AAA | CTC | ATC | TTT |  |  |  |  |  | 0 | 1 | 1 |  |  |  |  |  |
|  | TGT | GCC | TGT | GAC | ACG | TGG | GGA | TAC | ACC | GAT | AAA | CTC | ATC | TTT |  |  |  |  |  | 2 | 0 | 2 |  |  |  |  |  |

**Supplementary Table 2.** Sequences tested functionally in the TCR transfer experiments. The conserved cysteine of the V region and the phenylalanine of the J region were not included when calculating the CDR3 length.

| **TCR ID** | **Sequence** | **CDR3δ AA length** |
| --- | --- | --- |
|  | **Public sequences** |  |
| P1 | CACDT**L**GDTDKLIF(AJ3) | 12 |
| P2 | CACDV**L**GDTDKLIF(AJ1) | 12 |
| P3 | CACDT**V**GDTDKLIF(AJ4) | 12 |
| P4 | CACDT**W**GYTDKLIF(AJ2) | 12 |
|  | **Non-public sequences** |  |
| N1 | CACDT**L**LGIRQNTDKLIF(AN1) | 16 |
| N2 | CACDT**V**LGDPSYTDKLIF(AN3) | 16 |
| N3 | CACDT**V**GALGGTDKLIF(AN5) | 15 |
| N4 | CACDT**V**VPVGYTGGQKNDKLIF(AN6) | 20 |
| N5 | CACDT**V**GLITGGYSDDTDKLIF(AN7) | 20 |

**Supplementary Table 3.** Overhang PCR primer sequences used to generate CDR3δ of the functionally tested TCR chains.

| **TCR** | **R1** | | | **R2** | | |
| --- | --- | --- | --- | --- | --- | --- |
|  | **Forward Primer 5’** | **Reverse Primer 5’** | **Template** | **Forward Primer 5’** | **Reverse Primer 5’** | **Template** |
| P1 | TGCGACACACTCGGGGATACCGACAAGCTGATCTTCGGC | BamHI_rv ATGCGGATCCTCACAGG | pBullet_clone5_delta_puro | NcoI_fw  ACTGCCATGGAGCGGATCAGC | TCGGTATCCCCGAGTGTGTCGCAGGCGCAGTAGTAGC | pBullet_clone5_delta_puro |
| P2 | TGCGACGTCCTCGGGGACACCGACAAGCTGATCTTCGGC |  |  |  | TCGGTGTCCCCGAGGACGTCGCAGGCGCAGTAGTAGC |  |
| P3 | TGCGACACTGTCGGAGACACCGACAAGCTGATCTTCGGC |  |  |  | TCGGTGTCTCCGACAGTGTCGCAGGCGCAGTAGTAGC |  |
| P4 | TGCGACACTTGGGGGTACACCGACAAGCTGATCTTCGGC |  |  |  | TCGGTGTACCCCCAAGTGTCGCAGGCGCAGTAGTAGC |  |
| N1 | CTTCTGGGGATTCGCCAAAACACCGACAAGCTGATCTTCGGC |  |  |  | TTGGCGAATCCCCAGAAGGGTGTCGCAGGCGCAGTAGTAGC |  |
| N2 | TCTAGTATCCCCCAGTAGAAAAGGCCGGGTGTCGCAGGCGCAGTAGTAGC |  |  |  | GTACGATGGGTCCCCCAGTACGGTGTCGCAGGCGCAGTAGTAGC |  |
| N3 | GTGGGGGCTCTTGGGGGCACCGACAAGCTGATCTTCGGC |  |  |  | CCCAAGAGCCCCCACGGTGTCGCAGGCGCAGTAGTAGC |  |
| N4 | GTTTTGGCGAATCCCCAGAAGGGTGTCGCAGGCGCAGTAGTAGC |  |  |  | CCCCAGTATACCCCACGGGGACGACGGTGTCGCAGGCGCAGTAGTAGC |  |
| N5 | GGAGCTAGCGCCAGTTGGGTCGCAGGCGCAGTAGTAGC |  |  |  | CGAGTATCCCCCAGTAATGAGCCCCACGGTGTCGCAGGCGCAGTAGTAGC |  |

1. S. Ravens *et al.*, Microbial exposure drives polyclonal expansion of innate gammadelta T cells immediately after birth. *Proc Natl Acad Sci U S A* **117**, 18649-18660 (2020).

2. P. L. Ryan *et al.*, Heterogeneous yet stable Vdelta2(+) T-cell profiles define distinct cytotoxic effector potentials in healthy human individuals. *Proc Natl Acad Sci U S A* **113**, 14378-14383 (2016).

3. M. Yousfi Monod, V. Giudicelli, D. Chaume, M. P. Lefranc, IMGT/JunctionAnalysis: the first tool for the analysis of the immunoglobulin and T cell receptor complex V-J and V-D-J JUNCTIONs. *Bioinformatics* **20 Suppl 1**, i379-385 (2004).
